# Optimization of 3D Extrusion Printing Parameters for Raw and Extruded Dehulled Andean Fava Bean Flours Using Response Surface Methodology (RSM)

**DOI:** 10.3390/foods14050715

**Published:** 2025-02-20

**Authors:** Grimaldo Wilfredo Quispe Santivañez, Henry Juan Javier Ninahuaman, Joselin Paucarchuco Soto, Maria Teresa Pedrosa Silva Clerici, Rebeca Salvador-Reyes

**Affiliations:** 1 Escuela Profesional de Ingeniería Agroindustrial, Facultad de Ingeniería, Universidad Nacional Autónoma Altoandina de Tarma, Acobamba 120701, Peru; gquispe@unaat.edu.pe (G.W.Q.S.); hjavier@unaat.edu.pe (H.J.J.N.); 71083073@unaat.edu.pe (J.P.S.); 2 Departamento de Ciência de Alimentos e Nutrição, Universidade Estadual de Campinas (UNICAMP), São Paulo 13083-862, Brazil; mclerici@unicamp.br; 3 Facultad de Ingeniería, Universidad Tecnológica del Perú, Lima 150101, Peru

**Keywords:** 3D food printing, fava bean, *Vicia faba* L., thermoplastic extrusion, optimization, response surface methodology, printing accuracy, surface texture, ImageJ

## Abstract

This study optimizes the 3D extrusion printing parameters—water-to-flour ratio (X_1_), temperature (X_2_), and printing speed (X_3_)—for raw (RFB) and extruded (EFB) dehulled Andean fava bean flours to maximize print quality and minimize structural defects. A 2^3^ central composite design combined with response surface methodology (RSM) was used to identify the optimal conditions for achieving geometric precision, surface homogeneity, and textural stability. Physicochemical analyses showed that extrusion cooking substantially modified the composition and rheology of the flour. Compared with RFB, EFB exhibited lower protein and fiber contents, a higher proportion of digestible carbohydrates, and reduced rheological parameters (τ_0_, K, G′, G″), which facilitated printing. The evaluation of different parameter combinations revealed notable differences between the two flours, with X_1_ and X_2_ exerting the greatest influence on print quality. For RFB, the highest desirability (0.853) was achieved at X_1_ = 0.806, X_2_ = 23.18 °C, and X_3_ = 2470.5 mm/min, yielding more uniform and firmer printed structures. In contrast, EFB reached a desirability of 0.844 at X_1_ = 1.66 °C, X_2_ = 56.82 °C, and X_3_ = 1505.43 mm/min, indicating its outstanding geometric accuracy and robustness. In conclusion, raw flour requires higher hydration and lower temperatures to prevent excessive viscosity. In contrast, extruded flour benefits from low water and high temperatures to achieve stable structures and firm textures. These findings demonstrate the feasibility of using Andean fava bean flour in 3D food printing to create nutrient-dense, functional foods with improved printability. This work offers practical applications for developing personalized foods—such as customized meals for individuals with specific dietary requirements—while contributing to sustainable and secure food production. Future research should address long-term storage, post-printing drying methods, and scaling production.

## 1. Introduction

Three-dimensional printing is revolutionizing the food industry by offering various innovative applications with significant benefits. This technology allows for the creation of customized foods in terms of shape, texture, flavor, and nutritional value, adapting to the specific needs and preferences of consumers—including special diets, allergies, particular nutritional requirements, and specialized products for individuals with chewing and swallowing difficulties [1,2,3,4,5]. Recent advancements in 3D food printing (3DFP) have demonstrated its potential to address global challenges such as food security and sustainability by reducing food waste through precise ingredient usage and the incorporation of by-products, achieving waste reductions of up to 10–30% [6,7]. Furthermore, 3DFP enables the use of alternative protein sources, thereby contributing to a more sustainable food system [6]. In terms of personalized nutrition, 3DFP allows the creation of foods tailored to individual macronutrient and micronutrient requirements, with applications for populations such as the elderly, athletes, and patients [8]. The integration of artificial intelligence and big data can further optimize these solutions by analyzing individual needs [8]. The ability to create complex and personalized structures enhances food presentation and design, fostering creativity and flexibility in gastronomy while promoting the development of specialized food products tailored to diverse consumer needs [9,10].

In this context, the incorporation of legume flours into 3DFP further enhances its potential to develop nutrient-dense and functional foods. Legume flours are rich in proteins, fibers, and essential nutrients, making them ideal for enriching the nutritional profile of printed foods [11,12,13]. For example, adding legume flours to extruded rice products can reduce the glycemic index and improve their nutritional value [13]. Moreover, these flours can improve the texture and structural integrity of printed foods. It has been shown that whole flours, which include legume components, form strong gel networks that increase water-holding capacity and maintain a soft and elastic texture over time [14].

Recently, Andean fava beans (*Vicia faba* var. minor) have emerged as a potential superfood for innovation and nutritional improvement in foods [15]. They contain approximately 25% protein, with a high proportion of essential amino acids, and are rich in dietary fiber, minerals, and antioxidants, including polyphenols and flavonoids [16]. However, due to the presence of antinutrients in their composition—mainly in the husk—it is necessary to apply physical and thermal techniques to reduce their concentration. Antinutritional factors such as tannins, phytic acid, and vicine/convicine can reduce the bioavailability of essential nutrients and limit their digestibility if not adequately processed [17]. In Andean fava beans, the reported concentrations of phytic acid (0.56 mg/100 g), tannins (94.13 µg/g), and trypsin inhibitors (10.17 TIU/mg) demonstrate the need for effective processing strategies [18]. Techniques such as soaking, dehulling, and extrusion have proven effective in mitigating these compounds [19,20,21,22,23], although they can result in increased production costs and require careful optimization to balance nutritional improvements with economic feasibility, particularly for large-scale applications [24,25]. A recent study revealed that the combination of preprocessing techniques such as soaking and dehulling, followed by thermoplastic extrusion, increased protein digestibility, the bioaccessibility of bioactive compounds, and the formation of digestion-resistant peptides with antidiabetic and antihypertensive potential in Peruvian fava beans [18]. Additionally, these treatments are advantageous for their application in 3DFP because the removal of the husk reduces the content of insoluble fibers, which can hinder the uniform flow of food ink and affect print quality [26,27].

Thermoplastic extrusion is a thermal process that, while it may cause the loss of bioactive compounds and heat-sensitive nutrients, enhances the bioavailability and digestibility of key macronutrients such as carbohydrates and proteins [28,29,30,31]. It also modifies the rheological and hydrocolloid properties of legume flours [32,33], making them suitable for creating printable food inks. Extruded flours exhibit greater solubility and water absorption than their raw counterparts [34,35], and they have demonstrated good printability and shape stability in 3DFP. For example, mixtures of milk powder and rye flour better maintain their shape during baking than milk powder alone [36]. Similarly, composite flours of barnyard millet, green gram, and other ingredients were successfully used to produce fiber- and protein-rich snacks with good printability and consumer acceptance [37]. Despite these advantages, the use of extruded legume flours in 3DFP remains an underexplored field.

The composition and technological functionality—understood as the physicochemical and rheological properties that affect the hydration, flow, and structural behavior of flours during processing—are crucial for the success of 3DFP [38]. Studies have shown that the water–flour ratio significantly affects the printability and structural integrity of printed objects [39]. A higher rice flour content improves water retention, thereby increasing the printing capacity by maintaining shape and dimensional accuracy [40]. Additionally, thermomechanical treatment [12] and printing parameters are essential for achieving good print quality. Higher printing speeds (e.g., 20 mm/min) can improve material flow and reduce deformations, whereas lower speeds can lead to over-extrusion and greater deformations [41,42]. Temperature is another critical factor that directly affects the rheology and flow behavior of the food ink [40].

Response surface methodology (RSM) has been widely used in 3DFP processes, offering several advantages for improving the quality and functionality of printed products. For example, Liu et al. [43] optimized a mixture containing egg white protein, gelatin, corn starch, and sucrose to achieve the best rheological properties and sensory scores. Zhang et al. [44] optimized multicomponent inks fortified with alternative proteins and demonstrated that RSM can effectively reduce the number of experiments needed to investigate the interrelationships between ink composition and desired printing outcomes. Additionally, RSM has been employed to determine optimal printing parameters, such as the nozzle diameter, printing speed, and layer height, which significantly improves the printing quality and accuracy [45,46]. It has also been applied to predict and optimize the textural properties of 3DFP to ensure that they meet the desired sensory attributes [47,48].

In this context, it is essential to optimize the 3D printing parameters for Andean fava bean flours because this technology not only allows for modeling complex structures but also serves as a pre-processing stage that favorably modifies the functional and sensory properties of the final food. Andean fava beans, an emerging crop in Peru with high nutritional potential due to their protein and fiber content, remain underutilized in 3D food printing compared to other legumes and grains, such as chickpeas, lentils, and quinoa. This is largely due to limited studies on their printability and the lack of knowledge about key properties, such as their rheological behavior, which are critical for achieving structural accuracy and stability in printed products. Evaluating both raw and extruded flours is particularly important because extrusion significantly alters the functional and rheological properties, potentially enhancing printability. Addressing these knowledge gaps will help determine whether Andean fava beans can be effectively used in the formulation of nutritionally enhanced 3DFP products. Therefore, the present study aimed to determine the optimal parameters for 3D extrusion printing—specifically, printing temperature, printing speed, and dough moisture (water-to-flour ratio)—in raw and extruded dehulled Andean fava bean flours using a design based on RSM. The results seek to promote the use of local ingredients, such as Andean fava beans, in the formulation of food inks that contribute to the development of printed foods with greater nutritional benefits and sustainability.

## 2. Materials and Methods

### 2.1. Production and Characterization of Dehulled Andean Fava Bean Flours

The same raw and extruded dehulled fava bean flour samples presented by Salvador-Reyes et al. [18] were used. Briefly, Peruvian Andean fava beans (Verde variety) (Bells, Lima, Peru) were soaked in water at room temperature (21 °C) for 24 h, then dehulled and dried in an oven at 50 °C until reaching less than 10% moisture content. The dried dehulled beans were milled to produce raw dehulled fava bean flour (RFB). The RFB was then conditioned to 18% moisture and subjected to cooking by extrusion using a co-rotating twin-screw extruder (ZSK 30-Werner & Pfleiderer Corp, Ramsey, MN, USA). The processing parameters were set at T_1_ = 70 °C, T_2_ = 90 °C, T_3_ = 120 °C, and T_4_ = 150 °C for each barrel zone temperature; the screw rotation speed was 150 rpm; the feed rate was 300 g/min; and the die diameter was 4.8 mm. The lower temperatures (T_1_ and T_2_) were chosen based on the fava bean flour gelatinization temperature (70.65 °C) [18], ensuring proper starch gelatinization and protein denaturation, while the higher temperatures (T_3_ and T_4_) were selected to reduce heat-sensitive antinutritional factors, such as trypsin inhibitors and tannins, to ensure proper starch gelatinization and protein denaturation [49,50,51]. The screw speed and feed rate were selected through pretests to ensure continuous extrusion without interruptions or clogging.

The obtained extruded products were dried (moisture < 10%) and milled (Hamilton Beach HBH650R, Shenzhen, China) to obtain extruded dehulled fava bean flour (EFB). To standardize the particle sizes of both samples, the RFB and EFB were sieved through a 35-mesh sieve (<500 μm). The samples were packed in high-density polyethylene bags and refrigerated (4 °C) before the subsequent analyses.

In the physicochemical characterization of the samples, the proximate composition was determined following the official AACCI methodology [52], including moisture (44–15.02), protein (46–13.01, nitrogen conversion factor = 5.4), crude fat (30–25.01), dietary fiber (32–05.01), and total ash (08–16.01). Digestible carbohydrates were calculated by subtracting the protein, crude fat, dietary fiber, and total ash contents from the initial weight of the sample (on a dry basis). Additionally, color parameters (L*, a*, and b*) were measured in the CIELab system using a CR-400 Chroma Meter (Konica Minolta, Tokyo, Japan). The morphology and structure of the flour particles were observed using a SU8000 field-emission scanning electron microscope (SEM) (HITACHI, Tokyo, Japan). The samples were dispersed on aluminum stubs and coated with a 15-nanometer copper-tin layer. The operating conditions of the SEM were an electron beam current of 80 μA, a constant acceleration voltage of 1 kV, and a working distance of 8.2 mm. Images were captured at 500× magnification.

The techno-functional analysis of the samples included the determination of the hydration properties. The water solubility index (WSI) and water absorption index (WAI) were assessed as described by Anderson et al. [53]. Rheological properties were also evaluated following the protocol described by Lemus-Moncada et al. [54] using a HAAKE RheoStress 6000 rheometer (Thermo Scientific, Karlsruhe, Germany). Samples were placed between parallel plates (60 mm) at 25 °C and compressed to obtain a gap of 1 mm. The rheological results were measured after 5 min of equilibrium to reach a measurement temperature of 25 °C. Flow curves were measured as a function of shear rate (0.01–10 s^−1^) to determine the shear stress and apparent viscosity of the samples. The data were then fitted to the Herschel–Bulkley model (Equation (1)), and a frequency sweep analysis was performed in the range of 0.1–100 Hz to evaluate the viscoelastic parameters of the samples. The storage modulus (G′), loss modulus (G″), and loss tangent (tan δ = G″/G′) were determined.

(1) $$\tau =\tau_{0}+k{r\gamma }^{n}$$

where τ is the shear stress (Pa), τ_0o_ is the yield stress (Pa), γ· is the shear rate (s^−1^), *k* is the consistency index (Pa·s^n^), and *n* is the flow behavior [55]. 

### 2.2. Experimental Design

The effects of varying the printing parameters were analyzed using response surface methodology (RSM) with a 2^3^ rotatable central composite design (RCCD). The independent variables studied were the water-to-flour ratio (X_1_), printing temperature (X_2_), and printing speed (X_3_). A total of 15 experiments (8 factorial, 6 axial, and 1 central) were performed (Table 1). The ranges of each parameter were determined separately for RFB and EFB through pre-tests, selecting ranges that allowed material flow without clogging the machine during printing. RCCD was selected due to its balance between experimental efficiency and precision in modeling non-linear responses, making it ideal for optimizing processes such as 3DFP, where interactions between variables can have quadratic effects.

### 2.3. 3D Printing

The three-dimensional printing experiments were performed in triplicate using a Foodini 3D printer (Natural Machines, Barcelona, Spain) equipped with a volume syringe of 100 mL and a 1.5 mm diameter nozzle. Prior to printing, the pastes were prepared by mixing flour and hot water (100 °C) in proportions established for each experiment (Table 1). The mixture was manually homogenized using a laboratory spoon until no lumps were observed (approximately 1 min). A five-pointed star shape (Feret’s diameter 7 cm) available in the Foodini Creator software (Version 4.0.3) was selected for printing, with 5 layers of 3 mm height, an ingredient flow rate of 1.7, a filling factor of 1, and a line thickness of 1.4 mm. The temperature and printing speed were varied according to the experiment (Table 1).

### 2.4. Quality Evaluation of 3DFP Products

#### 2.4.1. Surface Color Distribution

Considering that printed samples may present surface color heterogeneities due to the extrusion technique and dough formulation, we opted to determine the surface color distribution via computerized image analysis following the protocol described by Salvador-Reyes et al. [16]. Immediately after printing, the products from each experiment were placed on a black surface inside a light box under 5400 K illumination. Images were captured using a 24.2 MP digital color camera (Sony α6000 with 16–50 mm lens, f/3.5–5.6, at 50 mm, Shinagawa, Tokyo) at 30 cm from the samples, set at ISO-100, with an f/6.3 aperture and an exposure time of 1/80 s. The white balance was adjusted and background removal was performed using Adobe Photoshop 2020. The images were saved in PNG format and subsequently analyzed using ImageJ software (Version 13.0.6.) with the Color Inspector 3D v. 2.3 plugin, which provided a table with the colors in the red, green, and blue (RGB) space detected in the samples and the percentage of each based on pixel frequency.

#### 2.4.2. Printing Accuracy

Quantitative indicators of precision were determined using images from the color analysis (Section 2.4.1) and the reference figure (geometric design model). Both images were imported into ImageJ, and alignment was performed using the scale adjustment and rotation tools to ensure coincidence in position, scale, and orientation. To determine areas of discrepancy (mm^2^), the two images (reference and printed) were superimposed by calculating the difference (Image Calculator). Geometric parameters including total area (mm^2^), total perimeter (mm), Feret’s diameter (maximum distance between two points along the contour) (mm), circularity, round, and solidity were also measured for each experiment.

#### 2.4.3. Surface Texture of the Print

The surface texture of the print was evaluated using the gray level co-occurrence matrix (GLCM) method by employing the GLCM_Texture.class plugin of ImageJ software. The images, which were previously adjusted to white and without a background during the color analysis (Section 2.4.1), were converted to grayscale (8 bits) to highlight texture information. Subsequently, brightness and contrast adjustments were applied to optimize the visibility of the variations in gray tones, allowing for a detailed and precise analysis of the surface characteristics. The parameters measured were angular second moment (ASM), defined as a measure of texture uniformity or homogeneity, where higher values indicate smoother and more consistent surfaces; inverse difference moment (IDM), defined as a measure of the local uniformity of pixel intensities, with higher values indicating fewer abrupt changes and smoother transitions between adjacent pixels; and entropy, which quantifies the randomness or complexity of the grayscale distribution, where lower values suggest a more ordered and predictable texture.

#### 2.4.4. Instrumental Texture Profile

Textural parameters, such as firmness, cohesiveness, elasticity, and brittleness, were recorded using a Belle texture analyzer (Agrosta Overseas, Serqueux, France) with an 18-millimeter diameter aluminum probe. The deformation level was 25% of the original sample height at a speed of 3 mm/s, using a 5 g force transducer.

### 2.5. Statistical Analysis

All tests were performed in triplicate. The statistical analysis of the data obtained from the experimental design was performed using Design Expert software (v 23.1) (Stat-Ease, Minneapolis, MN, USA). The effects of the independent variables, regression coefficients (R^2^), and response surfaces were analyzed at a significance level of 5%. Linear, quadratic, and cubic regression models were tested, and the most significant (*p* < 0.05) and best-fitting models (R^2^ > 0.70) were selected.

ANOVA was used to evaluate the effects of independent variables and their interactions on the responses within the experimental design. Model adequacy was assessed by examining residual plots (provided in the Supplementary Materials) to confirm normality and homogeneity of variance, as well as by checking the adjusted and predicted R^2^ values to ensure goodness of fit and predictive performance. Additionally, a lack-of-fit test was used to verify the validity of the selected models. To identify significant differences among the samples, a one-way ANOVA was applied, followed by multiple comparisons using the Scott-Knott test when statistically significant differences were observed (*p* < 0.05).

## 3. Results

### 3.1. Physicochemical and Techno-Functional Characteristics of Raw and Extruded Andean Fava Bean Flours

Thermoplastic extrusion altered the physical, rheological, and color properties of the flours (Table 2).

Regarding proximate composition, a significant reduction in protein content was observed in EFB compared with RFB. This reduction may be attributed to protein denaturation and possible Maillard reactions during extrusion, which affect the solubility and availability of essential amino acids [49,56]. The dietary fiber content also decreased in EFB (*p* < 0.05), consistent with previous studies reporting the degradation of insoluble fiber due to heat and mechanical shear during extrusion [56]. This is reflected in a significant increase in the fraction of digestible carbohydrates, potentially arising from starch gelatinization and the breakdown of complex polysaccharides, thus raising the proportion of available carbohydrates [49].

Although extrusion reduced the protein and dietary fiber content, these changes reflect structural transformation rather than simple losses. Protein denaturation and aggregation during extrusion may reduce solubility but enhance digestibility and bioavailability by exposing binding sites for enzymatic action [28]. In addition, extrusion reduces antinutritional factors, further improving protein quality and the availability of essential amino acids [57]. This improvement is particularly beneficial for increasing the nutritional value of protein-rich products. The decrease in fiber is mainly associated with a shift from insoluble to soluble fiber, as reported in other legume-based extruded products [58]. Soluble fibers improve water-holding capacity and contribute to gel formation, which are essential properties for achieving uniform extrusion and shape stability in 3D food printing [59]. The interaction between soluble fiber and gelatinized starch may also form complexes that improve the texture and viscosity of the food ink, thereby optimizing its functionality during extrusion [60]. However, an increase in digestible carbohydrates could elevate the glycemic index of final products, which should be considered in product formulation for specific dietary needs.

**Figure 1:**
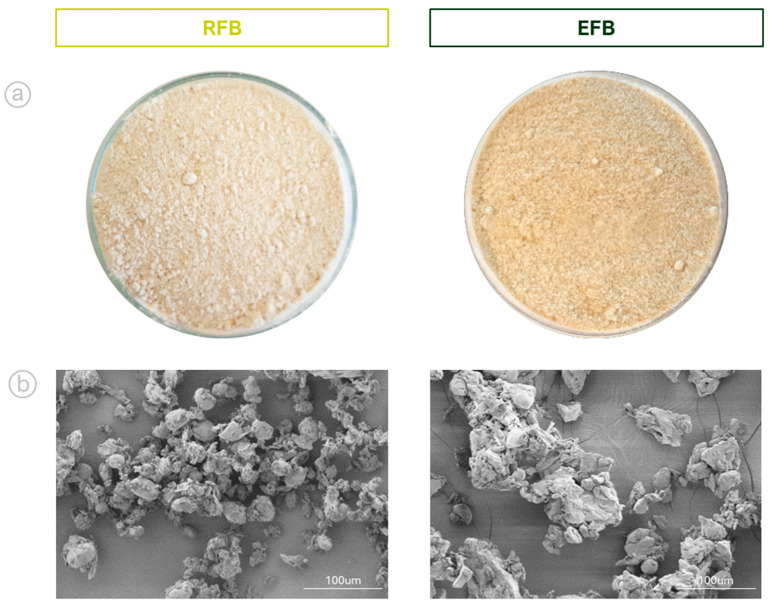
(**a**) Samples of RFB and EFB; (**b**) SEM images showing the morphology and particle size at 500× magnification.

Extrusion also significantly affected the color characteristics of the flour. A pronounced reduction in the L* value (95.10 vs. 83.59) was noted, indicating a darker product. Moreover, a* (0.57 vs. 3.81) and b* (6.57 vs. 23.58) increased significantly, indicating more intense reddish and yellowish hues, respectively. These alterations may stem from non-enzymatic browning reactions (e.g., the Maillard reaction) and partial degradation of natural pigments, in agreement with earlier findings for extruded legume products [61]. After extrusion, the initially native starch granules fused and formed larger complexes, as shown in Figure 1.

The rheological properties showed a significant decrease in yield stress (τ_0_), consistency index (*k*), and viscoelastic moduli (G′ and G″) in EFB compared to RFB (*p* < 0.05). For example, τ_0_ decreased from 52.64 Pa in RFB to 21.97 Pa in EFB, indicating a lower initial resistance to flow in the extruded flour. This reduction in τ_0_ facilitates the initiation of flow under low pressure, thus reducing the likelihood of clogging and ensuring smooth extrusion, which is beneficial for continuous 3D printing [62]. The consistency index (*k*) also decreased significantly from 507.97 to 359.82 Pa·s^n^, reflecting a lower shear resistance, allowing for a more uniform and continuous material flow through the nozzle [63]. The combination of reduced τ_0_ and *k* leads to better printability by minimizing interruptions during extrusion and promoting the deposition of consistent layers [64].

The storage modulus (G′) and loss modulus (G″) also decreased in the extruded flour, indicating a reduction in the material’s elastic and viscous properties. G′ decreased from 30,417.91 Pa in RFB to 23,969.48 Pa in EFB, while G″ decreased from 21,777.93 Pa to 15,679.52 Pa. Lower viscoelasticity enhances flow and material deformation, allowing the dough to adapt to the geometric constraints of the printing process while retaining sufficient structure upon deposition [65]. The tan δ ratio (G″/G′) slightly decreased from 0.72 in RFB to 0.65 in EFB (*p* < 0.01), indicating a more solid-like elastic behavior in the extruded flour. A lower tan δ suggests that the material is more elastic than viscous, which helps maintain the structural integrity of the printed object after extrusion [36,66].

These physicochemical, color, and rheological changes are crucial for understanding the behavior of extruded flour in various technological applications, including 3D food printing. In this context, texture, structural stability, printing accuracy, and color are key factors that influence product acceptance.

### 3.2. Quality of 3DFP Products

#### 3.2.1. Quality of Samples Printed with Raw Fava Bean Flour (RFB)

The quality evaluation of the samples printed using RFB revealed significant findings regarding their color surface (Table 3), printing accuracy, textural surface, and textural profile (Table 4). Increasing the water-to-flour ratio (X_1_) resulted in lighter yellow and golden tones, particularly in experiments where the ratio exceeded 0.75 (RFB-2, RFB-4, RFB-6, RFB-8, RFB-10, RFB-12, and RFB-13). Moreover, a higher water content led to a more homogeneous surface color distribution of samples, reflected by a decrease in the number of principal colors detected. These observations are consistent with previous reports indicating that adequate hydration promotes partial starch gelatinization and modification of the protein network, thereby generating a more uniform surface appearance [67]. Regarding temperature (X_2_), a slight intensification of yellowish or brownish hues was noted as the temperature rose from RFB-13 (23.18 °C), which exhibited a pale yellow color, to RFB-12 (56.82 °C), which showed a brighter yellow. This change could be primarily associated with enzymatic oxidations or partial protein transformations rather than the Maillard reaction because the latter typically requires higher temperatures (≥80–100 °C) or prolonged processing times to manifest strongly [68,69]. It is also possible that enzymes such as polyphenol oxidase and peroxidase remain partially active within this temperature range, contributing moderately to darkening [70,71]. Nevertheless, slight color changes may occur due to partial protein denaturation and the exposure of compounds susceptible to mild browning reactions at moderate temperatures [72]. In contrast, the printing speed (X_3_) did not significantly affect color distribution within the studied range, indicating that, at least for these speeds, the filament remained sufficiently stable during extrusion to maintain chromatic uniformity.

With respect to printing accuracy, increasing the water-to-flour ratio up to 0.8 and the temperature up to 40 °C reduced discrepancies in area, perimeter, and circularity, resulting in more regular shapes and closer adherence to the star design (Table 4). These results concur with studies on 3DFP matrices, which reported that a sufficient water content and moderate extrusion temperatures improve dimensional stability, decrease deformations, and enhance the cohesion of printed layers [73,74]. However, progressively increasing the printing speed caused greater distortion in some samples—for instance, RFB-4 showed layers displaced outside the intended print area—suggesting that once the speed surpasses a specific threshold, the extrusion and deposition of the material can no longer precisely follow the designed path, leading to defects in the final geometry [75].

Studies have indicated that moderate printing speeds are crucial for maintaining geometric accuracy. Specifically, speeds of 20–25 mm/s produced the best match to the target geometry in rice flour mixtures and baking dough constructs [41,76]. In contrast, higher speeds, such as 200 mm/s, are associated with increased porosity and structural instability, particularly in wheat flour-based matrices [77]. Additionally, increasing the speed from 10 mm/s to 70 mm/s significantly decreased the dimensional accuracy, as evidenced by a rise in the standard deviation of the measured dimensions [72]. These findings emphasize the need to optimize the printing speed to prevent deformations and ensure the structural integrity of 3DFP products.

Regarding surface texture, the GLCM results showed that higher water-to-flour ratios combined with lower temperatures (starting from 30 °C) and reduced printing speeds led to higher ASM values and lower entropy values. This implies greater homogeneity in the distribution of grayscale levels—concentrated in fewer combinations (high ASM)—along with lower randomness (low entropy), yielding more uniform surfaces and a more predictable texture [78]. The use of GLCM in food image analysis has become a robust tool for correlating surface structure with functional and processing properties because it helps identify uniformity or irregularity patterns that may be linked to changes in formulations and printing conditions [79,80,81]. The higher homogeneity (high ASM) and lower variability (low entropy) are due to hydration and the moderate temperature, which help form and stabilize the printed matrix. When the water-to-flour ratio is increased, starches are more likely to partially gelatinize, and proteins can restructure, producing more uniform surfaces that are less prone to irregularities in pixel intensity. Additionally, maintaining a moderate temperature (<30 °C) enhances the cohesion of the extruded material without causing drastic changes (e.g., extensive gelatinization or denaturation) that might produce visible heterogeneity in the printed layer. Consequently, grayscale levels tend to be concentrated in more specific combinations, raising the ASM, whereas lower entropy indicates a more orderly and consistent grayscale texture [67,73].

These surface texture improvements directly affect product appearance and consumer perception. Smoother and more homogeneous surfaces (high ASM, low entropy) enhance visual appeal because consumers often associate these attributes with higher product quality and better processing control [47]. Smooth textures enhance the perception of sweetness, which is particularly beneficial when paired with sweet-flavored products [47]. In contrast, rough or irregular surfaces can increase the perception of bitterness or intensity, which may be desirable for certain savory or bitter foods [82]. Additionally, rough textures in 3DFP have been linked to enhanced perceptions of saltiness, suggesting that surface texture could be strategically optimized to reduce the need for added salt in food formulations [83]. Based on our results, optimal printing parameters can be adjusted depending on whether RFB or EFB is selected, allowing for the development of surface textures that enhance flavor and improve consumer perception.

Regarding instrumental texture profile, the firmest and most cohesive samples were obtained using water-to-flour ratios below 0.67 and temperatures below 30 °C, as well as ratios above 0.75 and temperatures above 40 °C. This dual behavior could be attributed to the protein and carbohydrate content of fava bean flour. At lower water-to-flour ratios and temperatures, a denser matrix may form, resulting in a harder product. Conversely, at higher water ratios and slightly higher temperatures, proteins and starches achieve a degree of swelling and cross-linking that enhances cohesion and resistance to deformation [67,72].

**Figure 2:**
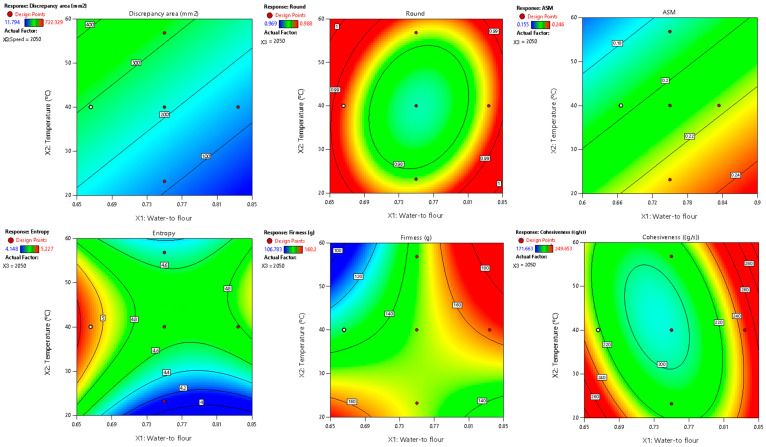
Two-dimensional contour plots of regression models for quality parameters of 3DFP samples with raw fava bean flour (RFB).

Among the parameters studied, area discrepancy, round, ASM, entropy, firmness, and cohesiveness produced satisfactory predictive models (R^2^ > 0.70) concerning the combined influence of the water-to-flour ratio, temperature, and printing speed (Table 5, Figure 2).

These models also passed the lack-of-fit test (*p* > 0.05), confirming their adequacy in representing the experimental data and minimizing unexplained variability. Additionally, normal probability and residual vs. predicted plots (provided in the Supplementary Materials) confirmed the normality and homogeneity of variance, further supporting the reliability of these models. Of these variables, the water-to-flour ratio and temperature exhibited the greatest impact, reflected in the highest coefficients within the mathematical models. Essentially, the water-to-flour ratio defines the dough’s rheology and shape-retention capacity, while temperature affects partial starch gelatinization, protein denaturation, and potential enzymatic reactions, thereby influencing texture and color. In contrast, printing speed had less influence under these conditions, likely because the values tested did not drastically compromise deposition or interlayer cohesion. Once stable extrusion was achieved at moderate speeds, changes in the deposition rate did not affect either the geometry or final homogeneity of the printed figures.

#### 3.2.2. Quality of Samples Printed with Extruded Fava Bean Flour (EFB)

The analysis of samples printed using EFB revealed a significant influence of the studied variables on color (Table 6) and overall printing quality (Table 7). Moreover, the geometric and technological properties of the samples were markedly improved compared to those produced with RFB. These differences are attributed to the extrusion process of the flour, in which heat and shear treatment promotes partial starch gelatinization, protein denaturation, and enzyme inactivation. Such changes in the structure and functionality of materials can lead to more stable and uniform printing [4,84].

The surface color distribution of the samples significantly varied depending on the water-to-flour ratio. At ratios below 0.70 (e.g., EFB-2, EFB-4, EFB-6, EFB-8, EFB-11), the figures displayed predominantly greenish and yellowish hues. These tones may be associated with phenolic compounds or residual pigments that are not fully inactivated during extrusion and become more concentrated at low water content. In contrast, at flour-to-water ratios above 0.70, the samples shifted toward yellow, brown, or reddish tones, which intensified slightly with increasing temperature (23.18 to 56.82 °C). This moderate darkening could be related to protein denaturation and mild browning reactions at intermediate temperatures, similar to those reported for extrusion and gentle thermal treatments [4,85]. As observed in the RFB formulations, printing speed (659 to 2341 mm/min) did not significantly affect surface color, suggesting that within this range, the deposition rate did not appreciably alter chromatic uniformity.

The printing accuracy was notably affected by the flour-to-water ratio and temperature. Samples printed at lower ratios and higher temperatures (e.g., EFB-3, EFB-7, EFB-11, EFB-12) or at higher ratios and moderate temperatures (e.g., EFB-2, EFB-6, EFB-13) showed reduced discrepancies in area, perimeter, and Feret diameter. These outcomes may be attributed to the stable rheological behavior of EFB, which includes lower yield stress (τ_0_ = 21.97 Pa) and consistency index (*k* = 359.82 Pa·s^n^) (Table 2). These parameters allow the material to flow in a controlled manner and remain cohesive during layer deposition, provided that the water-to-flour ratio is properly adjusted to achieve the necessary consistency and preserve the printed shape [86,87,88]. For instance, in cookie dough, yield stress values between 50.22 and 72.80 Pa are optimal for high printability and product stability [89]. As extruded flours exhibit greater water absorption capacity, a moderate temperature increase promotes water uptake and facilitates extrusion, achieving more precise layer deposition when operating at lower water-to-flour ratios and higher temperatures. Conversely, at lower temperatures, increasing the water content aids in producing a uniform material flow through the nozzle, which also leads to more well-defined shapes.

In these experiments, the circularity of the samples was lower, but their round and solidity values were higher than those of the central point. In the star-shaped design, the lower circularity indicates that the angular contours were better preserved than in the original design. Meanwhile, higher round and solidity values reflect fewer internal voids and, consequently, sturdier and more well-defined structures [90]. These findings are consistent with evidence that extrusion improves the molecular organization of proteins and starches, as well as their hydration and dough-forming properties, increasing cohesion and reducing susceptibility to deformation during 3DFP [91,92,93,94].

Surface texture analysis using GLCM revealed no significant differences (*p* > 0.05) between ASM and IDM across experiments. However, samples with higher water-to-flour ratios and printing speeds below 1500 mm/min (e.g., EFB-2, EFB-4, EFB-10, EFB-15) showed lower surface texture entropies. This result suggests that a higher water content promotes homogeneous layer fusion and fills microvoids, whereas moderate speeds allow for more stable material deposition without surface defects. Additionally, rising temperature was associated with a reduction in entropy (EFB-13 > EFB-9 > EFB-12), which may be explained by the slightly enhanced internal cohesion of the material at higher temperatures and more efficient water integration into the matrix [95,96].

The instrumental texture profile was influenced by the printing parameters. Samples printed at lower flour-to-water ratios and higher temperatures (e.g., EFB-2, EFB-6) displayed greater firmness, cohesiveness, elasticity, and brittleness than those printed at higher water ratios and lower temperatures (e.g., EFB-3, EFB-7). According to Table 2, EFB exhibits lower yield stress and viscoelastic moduli (G′ and G″), suggesting moderate elastic behavior that can become firmer under intermediate printing temperatures. This phenomenon promotes protein cross-linking and partial starch reorganization, providing greater mechanical strength to the product [72,97]. Additionally, the significant increase in digestible carbohydrates observed in EFB due to starch gelatinization and polysaccharide breakdown contributes to these textural changes. Gelatinized starches enhance matrix formation by absorbing water, increasing viscosity, and forming a denser gel network upon cooling, which reinforces the cohesion and firmness of the printed structure [98]. On the other hand, higher hydration at lower temperatures retains more moisture and reduces both firmness and structural cohesion by limiting starch retrogradation and protein cross-linking [99].

The printing speed (*p* < 0.05) also significantly affected the texture in the experiments. At lower speeds, the resulting products exhibited higher firmness, cohesiveness, elasticity, and brittleness, likely due to each layer having sufficient time to settle and partially cool, forming stronger interlayer bonds. For example, printing rice starch at speeds of 800–1500 mm/min yielded better printability and textural properties [100]. Likewise, in plant-based meat, lower printing speeds (40–80 mm/s) enhanced quality attributes such as hardness, elasticity, and chewiness [101]. In contrast, higher speeds can cause gaps or layer misalignments, thus undermining the overall mechanical integrity.

**Figure 3:**
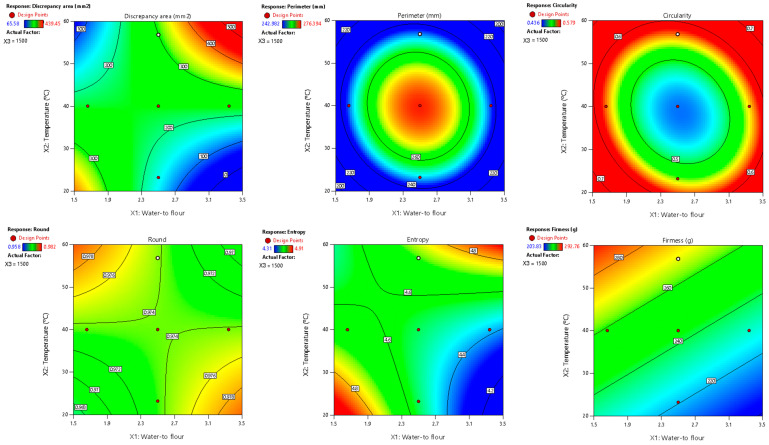
Two-dimensional contour plots of regression models for quality parameters of 3DFP samples with extruded fava bean flour (EFB).

Among the evaluated parameters, area discrepancy, perimeter, circularity, round, entropy, and firmness produced satisfactory predictive models (R^2^ > 0.70) regarding the combined influence of water-to-flour ratio, temperature, and printing speed (Table 8, Figure 3). These models also passed the lack-of-fit test (*p* > 0.05), confirming their adequacy in representing the experimental data and minimizing unexplained variability. Additionally, normal probability and residual vs. predicted plots (provided in the Supplementary Materials) confirmed the normality and homogeneity of variance, further supporting the reliability of these models. As in the RFB design, the water-to-flour ratio and temperature had the greatest impact, as evidenced by their highest coefficients in the mathematical models.

### 3.3. Optimization of Printing Parameters

Based on the predictive models obtained (R^2^ > 0.70), multiple-response optimization was performed for samples printed with both RFB and EFB. The objective was to minimize geometric inaccuracies—particularly area discrepancy—and to maximize surface texture homogeneity (reflected in roundness and entropy) as well as sample firmness, assigning equal importance to all responses (Table 9).

**Figure 4:**
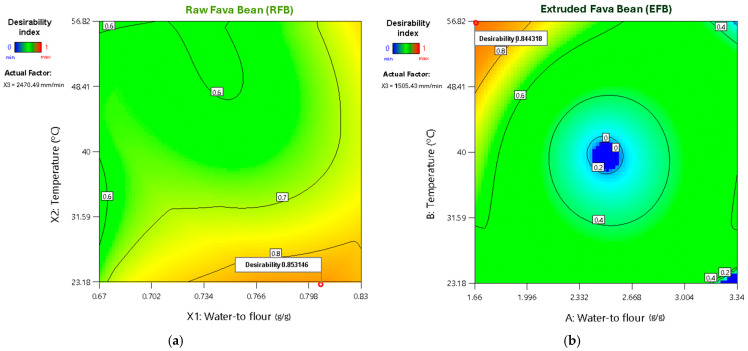
Two-dimensional contour plots of desirability parameter optimization for (**a**) RFB and (**b**) EFB.

For RFB, the highest desirability value (0.853) was obtained with a water-to-flour ratio of 0.806, a temperature of 23.18 °C, and a printing speed of 2470.5 mm/min after evaluating 92 possible solutions. In contrast, the maximum desirability of EFB (0.844) occurred at a water-to-flour ratio of 1.66, temperature of 56.82 °C, and printing speed of 1505.43 mm/min, following an assessment of 63 possible solutions. These results indicate that while RFB requires greater hydration and a lower printing temperature to achieve optimal prints, EFB is better suited to lower hydration and higher temperatures (Figure 4), yielding more precise shapes with homogeneous surfaces and a firmer texture.

These differences in printing requirements arise from the distinct physicochemical and rheological properties of each raw material (Table 2). In the case of RFB, its higher protein and fiber contents, coupled with elevated τ_0_, K, and viscoelastic moduli (G′ and G″), create a denser, more viscous network that requires more water and lower printing temperatures to avoid excessive denaturation or lump formation. Conversely, EFB contains a higher proportion of digestible carbohydrates and lower protein and fiber contents, as well as lower rheological parameters (τ_0_, K, G′, G″), facilitating easier initial flow with more elastic and fluid behavior. Under these conditions, EFB requires less water but requires a higher temperature to ensure sufficient cohesion and firmness during printing.

These findings have direct relevance for the emerging field of 3DFP, where controlling water content, temperature, and extrusion speed is critical for achieving desirable texture, shape fidelity, and nutritional profiles. Studies on protein-rich materials (e.g., fish surimi or plant-based inks) have similarly demonstrated that optimizing printing parameters can improve structural stability and consumer acceptability [102,103]. In practice, the ability to fine-tune dough properties by modifying hydration and processing temperature opens pathways for producing customized snack prototypes, personalized nutritionally enhanced products, and value-added functional foods using Andean fava bean flour. This aligns with broader trends in the food industry, where 3DFP is increasingly being explored for on-demand production of novel shapes, tailored textures, or specialty diets in healthcare and senior nutrition [104]. By showing how raw and extruded flours respond differently to printing conditions, our results lay the groundwork for adapting formulations to a variety of production scales and product requirements, potentially reducing waste and enhancing product diversity in the bakery, snack, and convenience food sectors.

## 4. Conclusions

Using RSM, this study successfully identified the optimal 3D extrusion printing parameters—printing temperature, printing speed, and dough moisture (water-to-flour ratio)—for raw (RFB) and extruded (EFB) dehulled Andean fava bean flours. The thermoplastic extrusion process significantly modified the physicochemical, rheological, and colorimetric characteristics of the flour, leading to notable differences in printing performance between RFB and EFB.

For RFB, the optimal conditions (water-to flour ratio of 0.806, temperature of 23.18 °C, and printing speed of 2470.5 mm/min) maximized the shape accuracy, textural uniformity (assessed via GLCM), and firmness while minimizing geometric discrepancies. In contrast, EFB showed its best performance under a lower water-to-flour ratio (1.66) but higher temperature (56.82 °C) and moderate printing speed (1505.43 mm/min), yielding precise geometry, homogeneous surfaces, and a robust texture. These differences underscore the importance of adjusting the printing parameters to each flour’s unique compositional and rheological profile.

Overall, the findings highlight the potential of Andean fava bean flours—particularly those subjected to extrusion—to develop nutritionally enhanced 3DFP products with desirable structural and sensory attributes. Scaling this technology to industrial production will require further investigation into the continuous extrusion of food pastes, optimization of large-scale printing devices, and control over potential batch-to-batch variability. A challenge in industrial settings involves maintaining consistent textures and print fidelity across different production scales. Additionally, extrusion can lead to partial nutrient loss, such as a reduction in thermolabile vitamins or bioactive peptides, which should be addressed through process optimization or post-extrusion fortification. Tailored processing strategies are essential to balance functional, sensory, and nutritional trade-offs, ensuring the commercial viability of 3DFP products while preserving their health benefits.

Future research should explore the application of these methods to other legumes, considering their varying starch and protein profiles, and conduct detailed sensory evaluations to assess consumer acceptance and flavor optimization.

## Figures and Tables

**Table 1:** Coded and actual values of the 2^3^ RCCD for experiments with raw (RFB) and extruded (EFB) fava bean flour.

Experiment	Point Type	Coded Values ^1^			Real Values		
		X_1_	X_2_	X_3_	Water-to Flour (g:g)	Temperature (°C)	Speed (mm/min)
Raw fava bean							
RFB-1	Factorial	(−1)	(−1)	(−1)	0.7	30	1800
RFB-2	Factorial	(+1)	(−1)	(−1)	0.8	30	1800
RFB-3	Factorial	(−1)	(+1)	(−1)	0.7	50	1800
RFB-4	Factorial	(+1)	(+1)	(−1)	0.8	50	1800
RFB-5	Factorial	(−1)	(−1)	(+1)	0.7	30	2300
RFB-6	Factorial	(+1)	(−1)	(+1)	0.8	30	2300
RFB-7	Factorial	(−1)	(+1)	(+1)	0.7	50	2300
RFB-8	Factorial	(+1)	(+1)	(+1)	0.8	50	2300
RFB-9	Central	0	0	0	0.75	40	2050
RFB-10	Axial	(+α)	0	0	0.83	40	2050
RFB-11	Axial	(−α)	0	0	0.67	40	2050
RFB-12	Axial	0	(+α)	0	0.75	56.82	2050
RFB-13	Axial	0	(−α)	0	0.75	23.18	2050
RFB-14	Axial	0	0	(+α)	0.75	40	2470.50
RFB-15	Axial	0	0	(−α)	0.75	40	1629.50
Extruded fava bean							
EFB-1	Factorial	(−1)	(−1)	(−1)	2	30	1000
EFB-2	Factorial	(+1)	(−1)	(−1)	3	30	1000
EFB-3	Factorial	(−1)	(+1)	(−1)	2	50	1000
EFB-4	Factorial	(+1)	(+1)	(−1)	3	50	1000
EFB-5	Factorial	(−1)	(−1)	(+1)	2	30	2000
EFB-6	Factorial	(+1)	(−1)	(+1)	3	30	2000
EFB-7	Factorial	(−1)	(+1)	(+1)	2	50	2000
EFB-8	Factorial	(+1)	(+1)	(+1)	3	50	2000
EFB-9	Central	0	0	0	2.5	40	1500
EFB-10	Axial	(+α)	0	0	3.34	40	1500
EFB-11	Axial	(−α)	0	0	1.66	40	1500
EFB-12	Axial	0	(+α)	0	2.5	56.82	1500
EFB-13	Axial	0	(−α)	0	2.5	23.18	1500
EFB-14	Axial	0	0	(+α)	2.5	40	2341
EFB-15	Axial	0	0	(−α)	2.5	40	659

^1^ α = 1.682 for the axial points in this three-variable Central Composite Rotatable Design (CCRD).

**Table 2:** Physicochemical and technological characteristics of raw (RFB) and extruded (EFB) Andean fava bean flours ^1^.

Characteristics	RFB	EFB	*p* < 0.05 ^2^
Centesimal composition (g/100 g)			
Protein	38.11 ± 1.11	33.74 ± 1.95	0.035 (**)
Fat	2.14 ± 0.04	2.06 ± 0.08	0.081 (*)
Ash	1.03 ± 0.05	1.03 ± 0.02	0.395 (*)
Dietary fiber	5.74 ± 0.19	4.52 ± 0.17	0.031 (**)
Digestible carbohydrates	50.98 ± 1.15	58.65 ± 2.02	0.015 (**)
Instrumental color			
L*	95.10 ± 0.06	83.59 ± 0.09	0.001 (**)
a*	0.57 ± 0.01	3.81 ± 0.08	0.001 (**)
b*	6.57 ± 0.79	23.58 ± 0.06	0.001 (**)
Rheology parameters			
τ_0_ (Pa)	52.64 ± 3.94	21.97 ± 1.94	0.005 (**)
K (Pa·s^n^)	507.97 ± 39.45	359.82 ± 29.65	0.040 (**)
*n*	0.48 ± 0.01	0.35 ± 0.02	0.120 (*)
R^2^	0.93 ± 0.06	0.95 ± 0.03	0.145 (*)
G′ (Pa)	30,417.91 ± 431.12	23,969.48 ± 319.48	0.007 (**)
G″ (Pa)	21,777.93 ± 314.15	15,679.52 ± 214.56	0.005 (**)
Tan δ	0.72 ± 0.03	0.65 ± 0.02	0.014 (**)

^1^ Initial moisture: RFB = 4.25 ± 0.25 (g/100 g), EFB = 4.97 ± 0.14 (g/100 g). ^2^ The asterisks in the last column indicate the presence (**) or absence (*) of significant differences between samples (*p* < 0.05) determined by Student’s *t*-test.

**Table 3:** Images and surface color distribution ^1^ of 3DFP samples printed with raw fava bean flour (RFB).

RFB-1	RFB-2	RFB-3	RFB-4	RFB-5
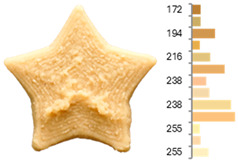	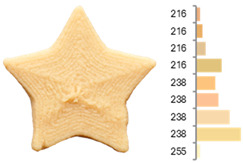	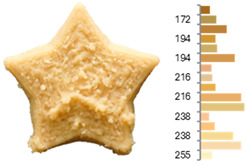	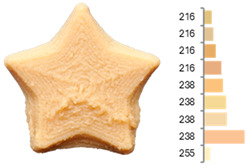	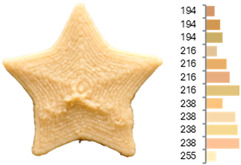
RFB-6	RFB-7	RFB-8	RFB-9	RFB-10
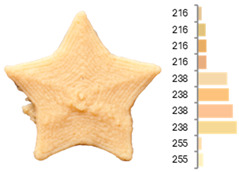	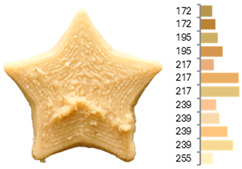	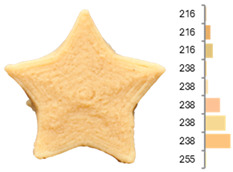	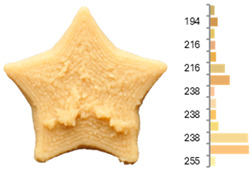	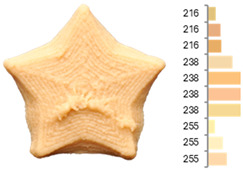
RFB-11	RFB-12	RFB-13	RFB-14	RFB-15
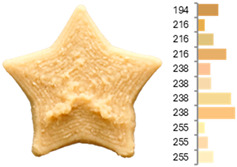	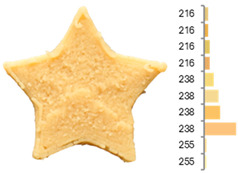	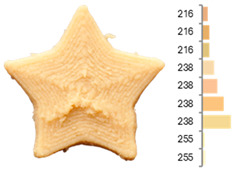	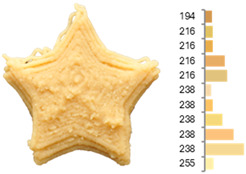	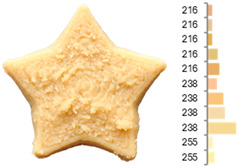

^1^ The colors shown next to the figures are presented in the RGB scale and represent the predominant colors (greater than 2% presence) on their surface.

**Table 4:** Printing accuracy and textural properties of 3DFP samples with RFB ^1^.

Experiments	Discrepancy Area (mm^2^)	Total Area (mm^2^)	Total Perimeter (mm)	Feret Diameter (mm)	Circularity	Round	Solidity	ASM ^2^	IDM ^3^	Entropy ^4^	Firmness (N)	Cohesiveness (g/s)	Elasticity (%)	Brittleness (%)
RFB-1	161.40 ^d^	2641.40 ^d^	265.70 ^b^	72.24 ^b^	0.47 ^d^	0.982 ^c^	0.77 ^c^	0.21 ^c^	0.66 ^ns^	4.799 ^b^	112.99 ^e^	171.66 ^e^	89.16 ^e^	65.32 ^e^
RFB-2	51.12 ^e^	2531.12 ^e^	249.17 ^d^	71.34 ^b^	0.51 ^c^	0.984 ^b^	0.75 ^c^	0.28 ^a^	0.63 ^ns^	4.421 ^c^	155.36 ^b^	212.70 ^b^	92.99 ^d^	67.34 ^c^
RFB-3	256.99 ^c^	2736.99 ^c^	253.64 ^c^	70.73 ^b^	0.54 ^b^	0.978 ^c^	0.76 ^c^	0.19 ^c^	0.64 ^ns^	5.025 ^a^	106.78 ^e^	183.30 ^c^	90.59 ^e^	66.24 ^d^
RFB-4	452.95 ^b^	2932.95 ^b^	243.82 ^e^	71.89 ^b^	0.62 ^a^	0.981 ^c^	0.85 ^b^	0.16 ^d^	0.60 ^ns^	5.227 ^a^	166.92 ^a^	227.10 ^b^	95.09 ^c^	68.58 ^b^
RFB-5	71.77 ^e^	2551.77 ^e^	257.08 ^c^	72.33 ^b^	0.49 ^d^	0.979 ^c^	0.74 ^c^	0.23 ^b^	0.66 ^ns^	4.562 ^c^	166.65 ^a^	249.65 ^a^	97.79 ^a^	71.05 ^a^
RFB-6	74.99 ^e^	2554.99 ^e^	260.85 ^b^	71.51 ^b^	0.47 ^d^	0.985 ^b^	0.75 ^c^	0.23 ^b^	0.64 ^ns^	4.406 ^c^	148.12 ^b^	226.99 ^b^	94.46 ^c^	68.38 ^b^
RFB-7	197.06 ^d^	2677.06 ^d^	256.82 ^c^	71.90 ^b^	0.51 ^c^	0.988 ^a^	0.77 ^c^	0.18 ^c^	0.69 ^ns^	4.587 ^c^	118.92 ^d^	192.60 ^c^	91.58 ^d^	66.78 ^c^
RFB-8	41.62 ^e^	2521.62 ^e^	248.89 ^d^	71.29 ^b^	0.61 ^a^	0.984 ^b^	0.76 ^c^	0.23 ^b^	0.66 ^ns^	4.213 ^d^	155.89 ^b^	223.31 ^b^	94.14 ^c^	68.49 ^b^
RFB-9	166.12 ^d^	2646.12 ^d^	247.77 ^d^	71.63 ^b^	0.55 ^b^	0.979 ^c^	0.79 ^c^	0.18 ^c^	0.63 ^ns^	4.823 ^b^	133.38 ^c^	192.66 ^c^	91.78 ^d^	66.81 ^c^
RFB-10	483.06 ^b^	2963.06 ^b^	243.85 ^e^	71.89 ^b^	0.63 ^a^	0.988 ^a^	0.78 ^c^	0.19 ^c^	0.65 ^ns^	4.678 ^c^	166.77 ^a^	239.11 ^a^	96.22 ^b^	68.79 ^b^
RFB-11	143.28 ^d^	2623.28 ^d^	247.40 ^d^	71.39 ^b^	0.54 ^b^	0.980 ^c^	0.86 ^b^	0.16 ^d^	0.60 ^ns^	5.136 ^a^	168.20 ^a^	242.38 ^a^	96.44 ^b^	69.43 ^b^
RFB-12	11.79 ^f^	2491.79 ^f^	260.57 ^b^	72.20 ^b^	0.54 ^b^	0.980 ^c^	0.78 ^c^	0.21 ^c^	0.66 ^ns^	4.798 ^b^	152.67 ^b^	210.26 ^b^	93.73 ^c^	67.86 ^b^
RFB-13	141.06 ^d^	2621.06 ^d^	265.78 ^b^	72.07 ^b^	0.46 ^d^	0.981 ^c^	0.61 ^d^	0.12 ^d^	0.70 ^ns^	4.148 ^d^	163.46 ^a^	223.22 ^b^	94.62 ^c^	68.23 ^b^
RFB-14	722.32 ^a^	3202.32 ^a^	322.15 ^a^	78.26 ^a^	0.47 ^d^	0.969 ^e^	0.77 ^c^	0.15 ^d^	0.65 ^ns^	4.727 ^b^	134.68 ^c^	193.47 ^c^	91.96 ^d^	66.78 ^c^
RFB-15	236.20 ^c^	2716.20 ^c^	248.30 ^d^	71.87 ^b^	0.39 ^e^	0.975 ^d^	0.93 ^a^	0.21 ^c^	0.65 ^ns^	4.725 ^b^	166.56 ^a^	215.77 ^b^	94.21 ^c^	67.93 ^b^

^1^ The letters next to the results indicate significant differences between the means according to the Scott-Knott test at *p* < 0.05. “ns” denotes non-significant differences. ^2^ ASM (angular second moment): This parameter reflects the uniformity or homogeneity of the pixel intensity distribution. Higher values indicate more uniform textures and consistent surface patterns. ^3^ IDM (inverse difference moment): This parameter measures texture smoothness by evaluating local variations. Higher IDM values suggest smoother textures with fewer abrupt changes. ^4^ Entropy: This parameter quantifies the randomness of the pixel intensity distribution. Lower entropy values denote more ordered, consistent, and predictable surface textures.

**Table 5:** Regression models of response surfaces for 3DFP quality parameters of RFB samples.

Dependent Variables	Regression Model	Lack of Fit (*p*-Value)	R^2^	R^2^ (Adjusted)
Discrepancy area (mm^2^)	1491.90 − 934.30 X_1_ + 5.91 X_2_ − 0.40 X_3_	0.2241	0.82	0.69
Round	1.72 − 2.07 X_1_ − 0.001 X_2_ + 0.002 X_3_ 0.002 X_1_X_2_ + 1.48 X_1_^2^ + 0.002 X_2_^2^	0.2849	0.79	0.18
ASM	0.10 + 0.122 X_1_ − 0.001 X_2_ + 0.0001 X_3_	0.3944	0.76	0.69
Entropy	19.09 − 53.36 X_1_ + 0.17 X_2_ − 0.003 X_3_ + 0.091 X_1_X_2_ − 0.003 X_1_X_3_ − 0.00001 X_2_X_3_ + 36.76 X_1_^2^ − 0.001 X_2_^2^ + 0.0003 X_3_^2^	0.4273	0.80	0.43
Firmness (g)	−972.24 + 1171.24 X_1_ − 9.21 X_2_ + 0.75 X_3_ + 18.32 X_1_X_2_ − 0.84 X_1_X_3_ − 0.002 X_2_X_3_	0.2682	0.78	0.66
Cohesiveness (g/s)	2199.49 − 7213.46 X_1_ − 6.10 X_2_ + 0.71 X_3_ + 14.03 X_1_X_2_ − 0.77 X_1_X_3_ − 0.004 X_2_X_3_ + 5580.40 X_1_^2^ − 0.05 X_2_^2^ + 0.0001 X_3_^2^	0.4487	0.80	0.45

**Table 6:** Images and surface color distribution ^1^ of 3DFP samples printed with extruded fava bean flour (EFB).

EFB-1	EFB-2	EFB-3	EFB-4	EFB-5
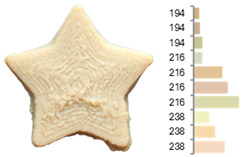	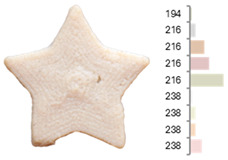	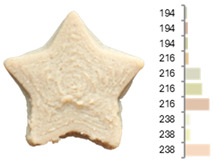	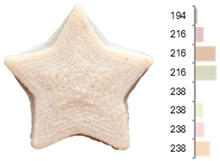	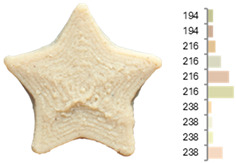
EFB-6	EFB-7	EFB-8	EFB-9	EFB-10
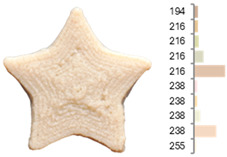	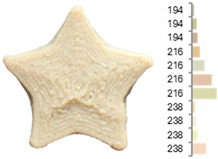	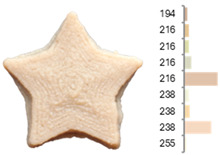	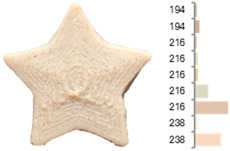	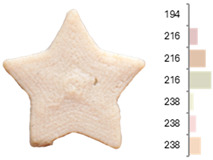
EFB-11	EFB-12	EFB-13	EFB-14	EFB-15
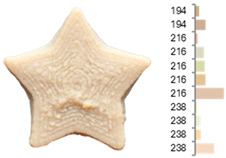	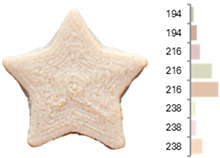	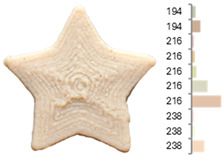	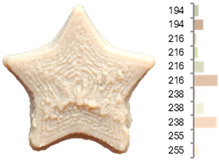	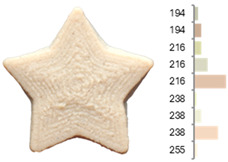

^1^ The colors shown next to the figures are presented in the RGB scale and represent the predominant colors (greater than 2% presence) on their surface.

**Table 7:** Printing accuracy and textural properties of 3DFP samples with EFB ^1^.

Experiments	Discrepancy Area (mm^2^)	Total Area (mm^2^)	Total Perimeter (mm)	Feret Diameter (mm)	Circularity	Round	Solidity	ASM ^2^	IDM ^3^	Entropy ^4^	Firmness (N)	Cohesiveness (g/s)	Elasticity (%)	Brittleness (%)
EFB-1	258.37 ^c^	2738.36 ^d^	252.74 ^c^	73.52 ^ns^	0.54 ^b^	0.958 ^e^	0.79 ^b^	0.20 ^ns^	0.62 ^ns^	4.76 ^b^	270.25 ^b^	338.46 ^d^	83.56 ^d^	62.57 ^c^
EFB-2	132.72 ^e^	2812.72 ^c^	247.12 ^d^	71.01 ^ns^	0.58 ^a^	0.970 ^d^	0.81 ^b^	0.18 ^ns^	0.61 ^ns^	4.25 ^d^	220.21 ^e^	302.03 ^f^	82.26 ^e^	61.19 ^d^
EFB-3	153.31 ^e^	2633.30 ^e^	251.08 ^c^	72.15 ^ns^	0.53 ^c^	0.976 ^c^	0.70 ^d^	0.23 ^ns^	0.62 ^ns^	4.67 ^c^	292.76 ^a^	373.69 ^a^	85.58 ^a^	64.55 ^a^
EFB-4	365.58 ^b^	2545.58 ^f^	244.957 ^f^	70.82 ^ns^	0.53 ^c^	0.969 ^d^	0.75 ^c^	0.22 ^ns^	0.65 ^ns^	4.32 ^d^	203.83 ^f^	289.67 ^f^	80.55 ^f^	59.33 ^e^
EFB-5	226.71 ^d^	2706.71 ^d^	253.73 ^b^	71.06 ^ns^	0.53 ^c^	0.973 ^d^	0.81 ^b^	0.20 ^ns^	0.61 ^ns^	4.91 ^a^	258.32 ^c^	351.34 ^c^	84.61 ^b^	63.40 ^b^
EFB-6	172.10 ^e^	2652.10 ^e^	276.39 ^a^	70.35 ^ns^	0.44 ^e^	0.982 ^a^	0.85 ^a^	0.20 ^ns^	0.62 ^ns^	4.71 ^b^	205.02 ^f^	295.33 ^f^	81.51 ^f^	60.64 ^d^
EFB-7	205.51 ^d^	2885.51 ^b^	252.03 ^c^	72.58 ^ns^	0.57 ^a^	0.973 ^d^	0.83 ^a^	0.17 ^ns^	0.62 ^ns^	4.87 ^b^	244.27 ^d^	309.16 ^e^	83.28 ^d^	62.18 ^c^
EFB-8	258.95 ^c^	2738.94 ^d^	274.88 ^a^	72.51 ^ns^	0.46 ^d^	0.972 ^d^	0.65 ^e^	0.21 ^ns^	0.62 ^ns^	4.72 ^b^	206.37 ^f^	294.97 ^f^	81.20 ^f^	60.01 ^e^
EFB-9	182.73 ^e^	2662.72 ^e^	242.98 ^f^	71.19 ^ns^	0.57 ^a^	0.981 ^a^	0.75 ^c^	0.20 ^ns^	0.62 ^ns^	4.74 ^b^	264.59 ^c^	353.27 ^c^	84.25 ^c^	63.35 ^b^
EFB-10	241.65 ^c^	2721.64 ^d^	250.34 ^c^	72.15 ^ns^	0.52 ^c^	0.974 ^d^	0.80 ^b^	0.22 ^ns^	0.65 ^ns^	4.21 ^e^	271.53 ^b^	363.60 ^b^	84.96 ^b^	63.80 ^b^
EFB-11	156.45 ^e^	2636.45 ^e^	255.47 ^b^	72.48 ^ns^	0.53 ^c^	0.971 ^d^	0.75 ^c^	0.21 ^ns^	0.61 ^ns^	4.42 ^c^	273.12 ^b^	343.12 ^d^	84.30 ^c^	63.62 ^b^
EFB-12	238.18 ^d^	2718.18 ^d^	244.66 ^d^	72.74 ^ns^	0.53 ^c^	0.977 ^b^	0.67 ^d^	0.21 ^ns^	0.61 ^ns^	4.61 ^c^	247.79 ^d^	322.57 ^e^	83.93 ^d^	63.00 ^c^
EFB-13	146.60 ^e^	2626.60 ^e^	255.06 ^b^	72.84 ^ns^	0.51 ^c^	0.979 ^b^	0.75 ^c^	0.22 ^ns^	0.62 ^ns^	4.84 ^a^	208.86 ^f^	301.20 ^f^	82.39 ^e^	61.44 ^d^
EFB-14	196.71 ^e^	2676.70 ^e^	247.60 ^d^	70.64 ^ns^	0.55 ^b^	0.978 ^b^	0.72 ^c^	0.20 ^ns^	0.61 ^ns^	4.98 ^a^	225.59 ^e^	309.84 ^e^	82.53 ^e^	61.57 ^d^
EFB-15	439.45 ^a^	2919.44 ^a^	243.89 ^f^	70.49 ^ns^	0.58 ^a^	0.975 ^c^	0.86 ^a^	0.19 ^ns^	0.61 ^ns^	4.35 ^d^	275.00 ^b^	340.63 ^d^	84.26 ^c^	63.39 ^b^

^1^ The letters next to the results indicate significant differences between the means according to the Scott-Knott test at *p* < 0.05. “ns” denotes non-significant differences. ^2^ ASM (angular second moment): This parameter reflects the uniformity or homogeneity of the pixel intensity distribution. Higher values indicate more uniform textures and consistent surface patterns. ^3^ IDM (inverse difference moment): This parameter measures texture smoothness by evaluating local variations. Higher IDM values suggest smoother textures with fewer abrupt changes. ^4^ Entropy: This parameter quantifies the randomness of the pixel intensity distribution. Lower entropy values denote more ordered, consistent, and predictable surface textures.

**Table 8:** Regression models of response surfaces for 3DFP quality parameters of EFB samples.

Dependent Variables	Regression Model	Lack of Fit *p*-Value	R^2^	R^2^ (Adjusted)
Discrepancy area (mm^2^)	1213.87 − 673.58 X_1_ − 11.28 X_2_ + 0.06 X_3_ + 12.36 X_1_X_2_ + 0.12 X_1_X_3_ − 0.01 X_2_X_3_	0.4211	0.77	0.42
Perimeter (mm)	−195.27 + 209.83 X_1_ + 8.32 X_2_ + 0.05 X_3_ − 0.23 X_1_X_2_ + 0.004 X_1_X_3_ − 0.0001 X_2_X_3_ − 41.68 X_1_^2^ − 0.10 X_2_^2^ − 0.0001 X_3_^2^	0.6175	0.86	0.61
Circularity	2.34 − 0.85 X_1_ − 0.03 X_2_ − 0.003 X_3_ +0.0002 X_1_X_2_ + 0.15 X_1_^2^ + 0.0003 X_2_^2^	0.2950	0.75	0.29
Round	0.86 + 0.02 X_1_ + 0.002 X_2_ + 0.0001 X_3_ + 0.003 X_1_X_2_	0.3438	0.82	0.74
Entropy	8.32 − 1.15 X_1_ − 0.05 X_2_ − 0.002 X_3_ + 0.016 X_1_X_2_ + 0.0001 X_1_X_3_	0.2487	0.73	0.44
Firmness (g)	259.07 − 16.97 X_1_ + 1.43 X_2_ − 0.019 X_3_	0.2239	0.70	0.32

**Table 9:** Selected parameters for desirability optimization and optimal responses of 3DFP samples with raw fava bean (RFB) and extruded fava bean (EFB) flours at the best desirability value.

Variables	Criterion	Lower Limit	Upper Limit	Importance	Expected Values
RFB					
X_1_: Water-to-flour ratio	In range	0.67	0.83	3	0.806
X_2_: Temperature (°C)	In range	23.18	56.82	3	23.18
X_3_: Speed (mm/min)	In range	1629.5	2470.5	3	2470.5
Discrepancy area (mm^2^)	Minimize	11	722.3	5	10.41
Round	Maximize	1	1	3	0.99
ASM	Maximize	0.2	0.3	3	0.247
Entropy	Minimize	4	5.2	3	4.275
Firmness (g)	Maximize	106.8	170	3	151.503
Cohesiveness (g/s)	Maximize	170	250	3	256.817
EFB					
X_1_: Water-to-flour ratio	In range	1.66	3.34	3	1.66
X_2_: Temperature (°C)	In range	23.18	56.82	3	56.82
X_3_: Speed (mm/min)	In range	659	2341	3	1505.43
Discrepancy area (mm^2^)	Minimize	65.6	439.4	5	36.20
Perimeter (mm)	Minimize	242	276	3	222.93
Circularity	Maximize	0.4	0.6	3	0.631
Round	Maximize	0.9	1	3	0.977
Entropy	Minimize	4.3	4.9	3	4.49
Firmness (g)	Maximize	203.8	292.8	3	277.75

## Data Availability

The original contributions presented in this study are included in the article/Supplementary Materials. Further inquiries can be directed to the corresponding author.

## Supplementary Materials

The following supporting information can be downloaded at: https://www.mdpi.com/article/10.3390/foods14050715/s1, Figure S1–S12: Normal Probability Plot and Residuals vs. Predicted Plot for Response in RFB and EFB printed samples.

## References

[1] Rogers H., Srivastava M. (2021). Emerging Sustainable Supply Chain Models for 3D Food Printing. Sustainability.

[2] Guan H., Sun Y., Liu X., Zhang Z. (2024). Research Progress of 3D Printing Technology in Animal-Derived Food Processing. Food Ferment. Ind..

[3] Donn P., Prieto M.A., Mejuto J.C., Cao H., Simal-Gandara J. (2022). Functional Foods Based on the Recovery of Bioactive Ingredients from Food and Algae By-Products by Emerging Extraction Technologies and 3D Printing. Food Biosci..

[4] Čukelj Mustač N., Pastor K., Kojić J., Voučko B., Ćurić D., Rocha J.M., Novotni D. (2023). Quality Assessment of 3D-Printed Cereal-Based Products. LWT.

[5] Besnea D., Avram M., Cananau S., Moraru E., Spanu A., Constantin V., Panait I.C. Constructive and Functional Particularities of 3D Printers with Applications in the Food Industry. Proceedings of the IOP Conference Series: Materials Science and Engineering.

[6] Padhiary M., Barbhuiya J.A., Roy D., Roy P. (2024). 3D Printing Applications in Smart Farming and Food Processing. Smart Agric. Technol..

[7] Mudau M., Adebo O.A. (2024). Three Dimensional (3D)-printed Foods: A Review of Recent Advances in Their Ingredients, Printing Techniques, Food Printers, Post-processing Methods, Consumer Acceptance and Safety. J. Food Process Eng..

[8] Thorakkattu P., Awasti N., Sajith Babu K., Khanashyam A.C., Deliephan A., Shah K., Singh P., Pandiselvam R., Nirmal N.P. (2025). 3D Printing: Trends and Approaches toward Achieving Long-Term Sustainability in the Food Industry. Crit. Rev. Biotechnol..

[9] Dancausa Millán M.G., Millán Vázquez de la Torre M.G. (2024). 3D Food Printing: Technological Advances, Personalization and Future Challenges in the Food Industry. Int. J. Gastron. Food Sci..

[10] Ulrikh E.V., Verkhoturov V.V. (2022). Features of Food Design on a 3d Printer. A Review. Pis. Sist. Food Syst..

[11] Raja V., Moses J.A., Anandharamakrishnan C. (2023). Effect of 3D Printing Conditions and Post-Printing Fermentation on Pearl Millet Fortified Idli. J. Sci. Food Agric..

[12] Guénard-Lampron V., Liu X., Masson M., Blumenthal D. (2023). Screening of Different Flours for 3D Food Printing: Optimization of Thermomechanical Process of Soy and Rye Flour Dough. Innov. Food Sci. Emerg. Technol..

[13] Guan C., Long X., Long Z., Lin Q., Liu C. (2023). Legumes Flour: A Review of the Nutritional Properties, Physiological Functions and Application in Extruded Rice Products. Int. J. Food Sci. Technol..

[14] Zheng L., Liu J., Liu R., Xing Y., Jiang H. (2021). 3D Printing Performance of Gels from Wheat Starch, Flour and Whole Meal. Food Chem..

[15] Salvador-Reyes R., Furlan L.C., Martínez-Villaluenga C., Dala-Paula B.M., Clerici M.T.P.S. (2023). From Ancient Crop to Modern Superfood: Exploring the History, Diversity, Characteristics, Technological Applications, and Culinary Uses of Peruvian Fava Beans. Food Res. Int..

[16] Salvador-Reyes R., Furlan L.C., Martínez-Villaluenga C., Martins Dala-Paula B., Harumi Nabeshima E., da Costa Pinto C., Michielon de Souza S., Azevedo Lima Pallone J., Teresa Pedrosa Silva Clerici M. (2024). Peruvian Fava Beans for Health and Food Innovation: Physicochemical, Morphological, Nutritional, and Techno-Functional Characterization. Food Res. Int..

[17] Mayer Labba I.C., Frøkiær H., Sandberg A.S. (2021). Nutritional and Antinutritional Composition of Fava Bean (*Vicia Faba* L., Var. Minor) Cultivars. Food Res. Int..

[18] Salvador-Reyes R., Teresa Pedrosa Silva Clerici M., Martínez-Villaluenga C. (2024). Enhancing the Nutritional and Bioactive Benefits of Faba Bean Flour by Combining Preprocessing and Thermoplastic Extrusion: A Comprehensive Study on Digestion-Resistant Peptides. Food Res. Int..

[19] Huma N., Anjum F.M., Sehar S., Issa Khan M., Hussain S. (2008). Effect of Soaking and Cooking on Nutritional Quality and Safety of Legumes. Nutr. Food Sci..

[20] Siah S., Wood J.A., Agboola S., Konczak I., Blanchard C.L. (2014). Effects of Soaking, Boiling and Autoclaving on the Phenolic Contents and Antioxidant Activities of Faba Beans (*Vicia Faba* L.) Differing in Seed Coat Colours. Food Chem..

[21] El-Hady E.A.A., Habiba R.A. (2003). Effect of Soaking and Extrusion Conditions on Antinutrients and Protein Digestibility of Legume Seeds. LWT Food Sci. Technol..

[22] Shi L., Arntfield S.D., Nickerson M. (2018). Changes in Levels of Phytic Acid, Lectins and Oxalates during Soaking and Cooking of Canadian Pulses. Food Res. Int..

[23] Shi D., Stone A.K., Marinangeli C.P.F., Carlin J., Nickerson M.T. (2024). Faba bean nutrition: Macronutrients, antinutrients, and the effect of processing. Cereal Chem..

[24] Patterson C.A., Curran J., Der T. (2017). Effect of Processing on Antinutrient Compounds in Pulses. Cereal Chem..

[25] Popova A., Mihaylova D. (2019). Antinutrients in Plant-Based Foods: A Review. Open Biotechnol. J..

[26] Brennan M.A., Monro J.A., Brennan C.S. (2008). Effect of Inclusion of Soluble and Insoluble Fibres into Extruded Breakfast Cereal Products Made with Reverse Screw Configuration. Int. J. Food Sci. Technol..

[27] Liu Y., Chen K., Zeng Q., Wang P., Zhang Y. (2025). The Impact of Dietary Fibers on the Construction and Molecular Network of Extrusion-Based 3D-Printed Chicken Noodles: Unlocking the Potential of Specialized Functional Food. Food Chem..

[28] Singh S., Gamlath S., Wakeling L. (2007). Nutritional Aspects of Food Extrusion: A Review. Int. J. Food Sci. Technol..

[29] Ek P., Ganjyal G.M., Ganjyal G.M. (2020). Basics of extrusion processing. Extrusion Cooking.

[30] Ek P., Kowalski R.J., Ganjyal G.M., Ganjyal G.M. (2020). Raw material behaviors in extrusion processing I (Carbohydrates). Extrusion Cooking.

[31] Alam M.S., Kaur J., Khaira H., Gupta K. (2016). Extrusion and Extruded Products: Changes in Quality Attributes as Affected by Extrusion Process Parameters: A Review. Crit. Rev. Food Sci. Nutr..

[32] Espinosa-Ramírez J., Rodríguez A., De la Rosa-Millán J., Heredia-Olea E., Pérez-Carrillo E., Serna-Saldívar S.O. (2021). Shear-Induced Enhancement of Technofunctional Properties of Whole Grain Flours through Extrusion. Food Hydrocoll..

[33] Comettant-Rabanal R., Carvalho C.W.P., Ascheri J.L.R., Chávez D.W.H., Germani R. (2021). Extruded Whole Grain Flours and Sprout Millet as Functional Ingredients for Gluten-Free Bread. LWT.

[34] Kaur G., Sharma S., Singh B., Dar B.N. (2016). Comparative Study on Functional, Rheological, Thermal, and Morphological Properties of Native and Modified Cereal Flours. Int. J. Food Prop..

[35] Martínez M.M., Rosell C.M., Gómez M. (2014). Modification of Wheat Flour Functionality and Digestibility through Different Extrusion Conditions. J. Food Eng..

[36] Lille M., Kortekangas A., Heiniö R.-L., Sozer N. (2020). Structural and Textural Characteristics of 3D-Printed Protein- and Dietary Fibre-Rich Snacks Made of Milk Powder and Wholegrain Rye Flour. Foods.

[37] Krishnaraj P., Anukiruthika T., Choudhary P., Moses J.A., Anandharamakrishnan C. (2019). 3D Extrusion Printing and Post-Processing of Fibre-Rich Snack from Indigenous Composite Flour. Food Bioproc. Technol..

[38] Wang M., Li D., Zang Z., Sun X., Tan H., Si X., Tian J., Teng W., Wang J., Liang Q. (2021). 3D Food Printing: Applications of Plant-Based Materials in Extrusion-Based Food Printing. Crit. Rev. Food Sci. Nutr..

[39] Masbernat L., Berland S., Leverrier C., Moulin G., Michon C., Almeida G. (2021). Structuring Wheat Dough Using a Thermomechanical Process, from Liquid Food to 3D-Printable Food Material. J. Food Eng..

[40] Başoğlu E.İ., Özgeçen A.B., Yavuz N. (2024). Enhancement of 3D-Printability of Zucchini Puree by Rice Flour Addition. Int. J. Food Sci. Technol..

[41] Thangalakshmi S., Arora V.K., Prithviraj V. (2022). Printability Assessment and Optimization of Process Parameters for 3D Printing of Rice Flour and Jaggery Paste. J. Biosyst. Eng..

[42] Barrios-Rodríguez Y.F., Igual M., Martínez-Monzó J., García-Segovia P. (2024). Multivariate Evaluation of the Printing Process on 3D Printing of Rice Protein. Food Res. Int..

[43] Liu L., Yang X., Bhandari B., Meng Y., Prakash S. (2020). Optimization of the Formulation and Properties of 3D-Printed Complex Egg White Protein Objects. Foods.

[44] Zhang Y., Lee A.Y., Pojchanun K., Lee C.P., Zhou A., An J., Hashimoto M., Tan U.-X., Leo C.H., Wong G. (2022). Systematic Engineering Approach for Optimization of Multi-Component Alternative Protein-Fortified 3D Printing Food Ink. Food Hydrocoll..

[45] Su A., He A., Ma G., Zhao L., Yang W., Hu Q. (2024). Modeling and Optimization of 3D Printing Process of Pleurotus Eryngii Powder Using Neural Network-Genetic Algorithm. Sci. Agric. Sin..

[46] Xia G., Tao L., Zhang S., Hao X., Ou S. (2024). An Optimization Study of 3D Printing Technology Utilizing a Hybrid Gel System Based on Astragalus Polysaccharide and Wheat Starch. Processes.

[47] Lee C.P., Hashimoto M. (2024). Prediction of Textural Properties of 3D-Printed Food Using Response Surface Methodology. Heliyon.

[48] Diañez I., Gallegos C., Brito-de la Fuente E., Martínez I., Valencia C., Sánchez M.C., Diaz M.J., Franco J.M. (2019). 3D Printing in Situ Gelification of κ-Carrageenan Solutions: Effect of Printing Variables on the Rheological Response. Food Hydrocoll..

[49] Alonso R., Aguirre A., Marzo F. (2000). Effects of Extrusion and Traditional Processing Methods on Antinutrients and in Vitro Digestibility of Protein and Starch in Faba and Kidney Beans. Food Chem..

[50] Sahni P., Sharma S. (2020). Influence of Processing Treatments on Cooking Quality, Functional Properties, Antinutrients, Bioactive Potential and Mineral Profile of Alfalfa. LWT.

[51] Das G., Sharma A., Sarkar P.K. (2022). Conventional and Emerging Processing Techniques for the Post-Harvest Reduction of Antinutrients in Edible Legumes. Appl. Food Res..

[52] American Association of Cereal Chemists (2000). Approved Methods of the American Association of Cereal Chemists.

[53] Anderson R.A., Conway H.F., Peplinski A.J. (1970). Gelatinization of Corn Grits by Roll Cooking, Extrusion Cooking and Steaming. Starch-Stärke.

[54] Lemus-Mondaca R., Puente-Díaz L., Vásquez-Montaño A., León E., Zura-Bravo L., Ortiz-Viedma J. (2024). Printability and Thermophysical Properties of Three-Dimensional-Printed Food Based on “Cochayuyo” Durvillaea Antarctica Seaweed Flour. Foods.

[55] Liu Y., Tang T., Duan S., Qin Z., Li C., Zhang Z., Liu A., Wu D., Chen H., Han G. (2020). Effects of Sodium Alginate and Rice Variety on the Physicochemical Characteristics and 3D Printing Feasibility of Rice Paste. LWT.

[56] Berrios J.D.J., Morales P., Cámara M., Sánchez-Mata M.C. (2010). Carbohydrate Composition of Raw and Extruded Pulse Flours. Food Res. Int..

[57] Li X., Manickavasagan A., Lim L.-T. (2024). Reduction of Antinutrients and Off-Flavour in Kidney Bean Flour by Acidic and Alkaline Reactive Extrusion. Food Res. Int..

[58] Naumann S., Schweiggert-Weisz U., Martin A., Schuster M., Eisner P. (2021). Effects of Extrusion Processing on the Physiochemical and Functional Properties of Lupin Kernel Fibre. Food Hydrocoll..

[59] Arshad R., Saqib A., Sharif H.R., Liaqat A., Xu B. (2025). Recent Advances in 3D Food Printing: Therapeutic Implications, Opportunities, Potential Applications, and Challenges in the Food Industry. Food Res. Int..

[60] Zhuang J., Zhu J., Cheung P.C.K., Li C. (2024). The Physical and Chemical Interactions between Starch and Dietary Fiber: Their Impact on the Physicochemical and Nutritional Properties of Starch. Trends Food Sci. Technol..

[61] Rocha-Guzman N.E., Gallegos-Infante J.A., Gonzalez-Laredo R.F., Bello-Perez A., Delgado-Licon E., Ochoa-Martinez A., Prado-Ortiz M.J. (2008). Physical Properties of Extruded Products from Three Mexican Common Beans (*Phaseolus Vulgaris* L.) Cultivars. Plant Foods Hum. Nutr..

[62] Agarwal D., Wallace A., Kim E.H.J., Wadamori Y., Feng L., Hedderley D., Morgenstern M.P. (2022). Rheological, Structural and Textural Characteristics of 3D-Printed and Conventionally-Produced Gluten-Free Snack Made with Chickpea and Lupin Flour. Future Foods.

[63] Chen J., Sun H., Mu T., Blecker C., Richel A., Richard G., Jacquet N., Haubruge E., Goffin D. (2022). Effect of Temperature on Rheological, Structural, and Textural Properties of Soy Protein Isolate Pastes for 3D Food Printing. J. Food Eng..

[64] Liu Z., Zhang M., Bhandari B., Yang C. (2018). Impact of Rheological Properties of Mashed Potatoes on 3D Printing. J. Food Eng..

[65] Zhu S., Stieger M.A., van der Goot A.J., Schutyser M.A.I. (2019). Extrusion-Based 3D Printing of Food Pastes: Correlating Rheological Properties with Printing Behaviour. Innov. Food Sci. Emerg. Technol..

[66] Siacor F.D.C., Chen Q., Zhao J.Y., Han L., Valino A.D., Taboada E.B., Caldona E.B., Advincula R.C. (2021). On the Additive Manufacturing (3D Printing) of Viscoelastic Materials and Flow Behavior: From Composites to Food Manufacturing. Addit. Manuf..

[67] Gholamipour-Shirazi A., Norton I.T., Mills T. (2019). Designing Hydrocolloid Based Food-Ink Formulations for Extrusion 3D Printing. Food Hydrocoll..

[68] Sui X., Meng Z., Dong T., Fan X., Wang Q. (2023). Enzymatic Browning and Polyphenol Oxidase Control Strategies. Curr. Opin. Biotechnol..

[69] Salvador-Reyes R., Paucar-Menacho L.M. (2019). Optimization of the Blanching Time and Temperature in the Manufacture of Hass Avocado Pulp Using Low Quality Discarded Fruits. Braz. J. Food Technol..

[70] Michalczyk D.J., Krupka M., Kamiński J., Wierzbicka M., Floryańska S., Kopeć W., Piotrowicz-Cieślak A.I. (2023). Physiological and Biochemical Parameters of Field Bean (*Vicia faba* Var. minor) Seeds Stored for 33 Years. Agriculture.

[71] Nogales-Delgado S. (2021). Polyphenoloxidase (Ppo): Effect, Current Determination and Inhibition Treatments in Fresh-Cut Produce. Appl. Sci..

[72] Liu Z., Zhang M., Bhandari B., Wang Y. (2017). 3D Printing: Printing Precision and Application in Food Sector. Trends Food Sci Technol..

[73] Zhang L., Lou Y., Schutyser M.A.I. (2018). 3D Printing of Cereal-Based Food Structures Containing Probiotics. Food Struct..

[74] Godoi F.C., Prakash S., Bhandari B.R. (2016). 3d Printing Technologies Applied for Food Design: Status and Prospects. J. Food Eng..

[75] Sun J., Zhou W., Huang D., Fuh J.Y.H., Hong G.S. (2015). An Overview of 3D Printing Technologies for Food Fabrication. Food Bioproc. Tech..

[76] Yang F., Zhang M., Fang Z., Liu Y. (2019). Impact of Processing Parameters and Post-Treatment on the Shape Accuracy of 3D-Printed Baking Dough. Int. J. Food Sci. Technol..

[77] Derossi A., Paolillo M., Caporizzi R., Severini C. (2020). Extending the 3D Food Printing Tests at High Speed. Material Deposition and Effect of Non-Printing Movements on the Final Quality of Printed Structures. J. Food Eng..

[78] Haralick R.M., Dinstein I., Shanmugam K. (1973). Textural Features for Image Classification. IEEE Transactions on Systems, Man, and Cybernetics.

[79] Baigts-Allende D., Ramírez-Rodrígues M., Rosas-Romero R. (2022). Monitoring of the Dehydration Process of Apple Snacks with Visual Feature Extraction and Image Processing Techniques. Appl. Sci..

[80] Davidovic L.M., Cumic J., Dugalic S., Vicentic S., Sevarac Z., Petroianu G., Corridon P., Pantic I. (2021). Gray-Level Co-Occurrence Matrix Analysis for the Detection of Discrete, Ethanol-Induced, Structural Changes in Cell Nuclei: An Artificial Intelligence Approach. Microsc. Microanal..

[81] Kristianto Y., Wignyanto W., Argo B.D., Santoso I. Changes of Gray Level Co-Occurrence Matrix (GLCM) Texture and Antioxidant Level during Freezing of Pumpkin. Proceedings of the IOP Conference Series: Earth and Environmental Science.

[82] van Rompay T.J.L., Finger F., Saakes D., Fenko A. (2017). “See Me, Feel Me”: Effects of 3D-Printed Surface Patterns on Beverage Evaluation. Food Qual. Prefer.

[83] van Rompay T., van Ooijen I., Groothedde S., Saakes D. (2021). (Not to Be Taken) with a Grain of Salt: Enhancing Perceived Saltiness by 3D-Printed Surface Textures. Food Qual. Prefer.

[84] Parmar P., Bobade H., Singh B., Pathania S. (2020). Extrusion Technologies for Cereal-Pulses Blends. Pulse Foods: Processing, Quality and Nutraceutical Applications.

[85] Kathuria D., Hamid, Gautam S., Thakur A. (2023). Maillard Reaction in Different Food Products: Effect on Product Quality, Human Health and Mitigation Strategies. Food Control.

[86] Chakraborty P., Eqbal M.D., Ahmed J. (2023). Three-Dimensional Printing and Its Application to Legume Proteins: A Review. Legume Sci..

[87] Liu Z., Bhandari B., Zhang M. (2019). Incorporation of Probiotics (*Bifidobacterium Animalis* Subsp. Lactis) into 3D Printed Mashed Potatoes: Effects of Variables on the Viability. Food Res. Int..

[88] Prithviraj V., Thangalakshmi S., Arora V.K., Liu Z. (2022). Characterization of Rice Flour and Pastes with Different Sweeteners for Extrusion-Based 3D Food Printing. J. Texture Stud..

[89] In J., Jeong H., Min S.C. (2022). Material Requirements for Printing Cookie Dough Using a Fused Deposition Modeling 3D Printer. Food Sci. Biotechnol..

[90] Huang M.s, Zhang M., Bhandari B. (2019). Assessing the 3D Printing Precision and Texture Properties of Brown Rice Induced by Infill Levels and Printing Variables. Food Bioproc. Technol..

[91] Téllez-Morales J.A., Herman-Lara E., Gómez-Aldapa C.A., Rodríguez-Miranda J. (2020). Techno-Functional Properties of the Starch-Protein Interaction during Extrusion-Cooking of a Model System (Corn Starch and Whey Protein Isolate). LWT.

[92] Zhang H., Meng Y., Liu X., Guan X., Huang K., Li S. (2019). Effect of Extruded Mung Bean Flour on Dough Rheology and Quality of Chinese Noodles. Cereal Chem..

[93] Mohamed I.O. (2023). Interaction of Starch with Some Food Macromolecules during the Extrusion Process and Its Effect on Modulating Physicochemical and Digestible Properties. A Review. Carbohydr. Polym. Technol. Appl..

[94] Shi M., Dong X., Cheng Y., Ji X., Liu Y., Yan Y. (2023). Preparation and Characterization of Extruded Yam Starch–Soy Protein Isolate Complexes and Their Effects on the Quality of Dough. Foods.

[95] Ma Y., Schutyser M.A.I., Boom R.M., Zhang L. (2022). Thermographic and Rheological Characterization of Viscoelastic Materials for Hot-Extrusion 3D Food Printing. Innov. Food Sci. Emerg. Technol..

[96] Liu Z., Bhandari B., Prakash S., Mantihal S., Zhang M. (2019). Linking Rheology and Printability of a Multicomponent Gel System of Carrageenan-Xanthan-Starch in Extrusion Based Additive Manufacturing. Food Hydrocoll..

[97] Wang Y., Zhang L., Cao G., Li Z., Du M. (2024). Effect of Heat Treatment on Gelatin Properties and the Construction of High Internal Phase Emulsions for 3D Printing. Foods.

[98] Gong Y., Xiao S., Yao Z., Deng H., Chen X., Yang T. (2024). Factors and Modification Techniques Enhancing Starch Gel Structure and Their Applications in Foods:A Review. Food Chem. X.

[99] Liu X., Chao C., Yu J., Copeland L., Wang S. (2021). Mechanistic Studies of Starch Retrogradation and Its Effects on Starch Gel Properties. Food Hydrocoll..

[100] Theagarajan R., Moses J.A., Anandharamakrishnan C. (2020). 3D Extrusion Printability of Rice Starch and Optimization of Process Variables. Food Bioproc. Tech..

[101] Cai W., Wang Y., Gao Y., Li L. (2023). Optimization of the Process Parameters for 3D Printing Plant Protein Meat. Nongye Gongcheng Xuebao/Trans. Chin. Soc. Agric. Eng..

[102] Pulatsu E., Su J.W., Lin J., Lin M. (2020). Factors Affecting 3D Printing and Post-Processing Capacity of Cookie Dough. Innov. Food Sci. Emerg. Technol..

[103] Wang L., Zhang M., Bhandari B., Yang C. (2018). Investigation on Fish Surimi Gel as Promising Food Material for 3D Printing. J. Food Eng..

[104] Dick A., Bhandari B., Prakash S. (2019). 3D Printing of Meat. Meat Sci..

