# Supplementary material for: Optimization of 3D Extrusion Printing Parameters for Raw and Extruded Dehulled Andean Fava Bean Flours Using Response Surface Methodology (RSM)
Source: Foods. 2025 Feb 20;14(5):715. doi: 10.3390/foods14050715 (PMC11899543; doi:10.3390/foods14050715)

Figure S1. Normal Probability Plot and Residuals vs. Predicted Plot for the Discrepancy Area Response in RFB printed samples

Response: Discrepancy area

Color points by value:

Discrepancy area:

11.794 722.329

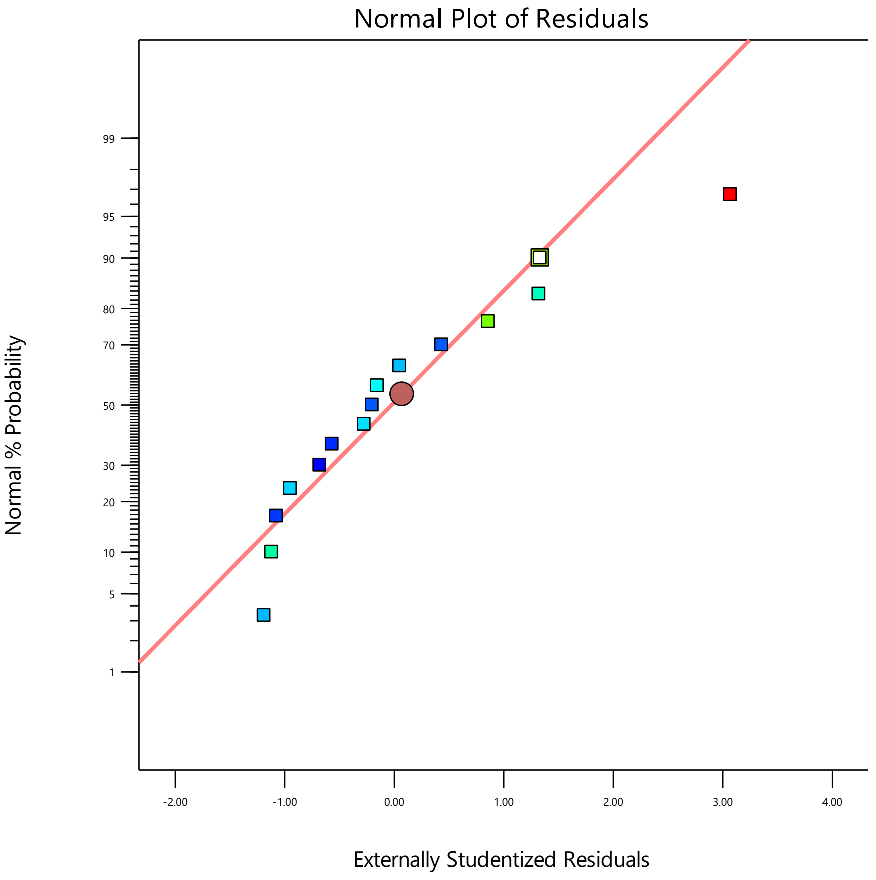

Response: Discrepancy area

Color points by value:

Discrepancy area:

11.794 722.329

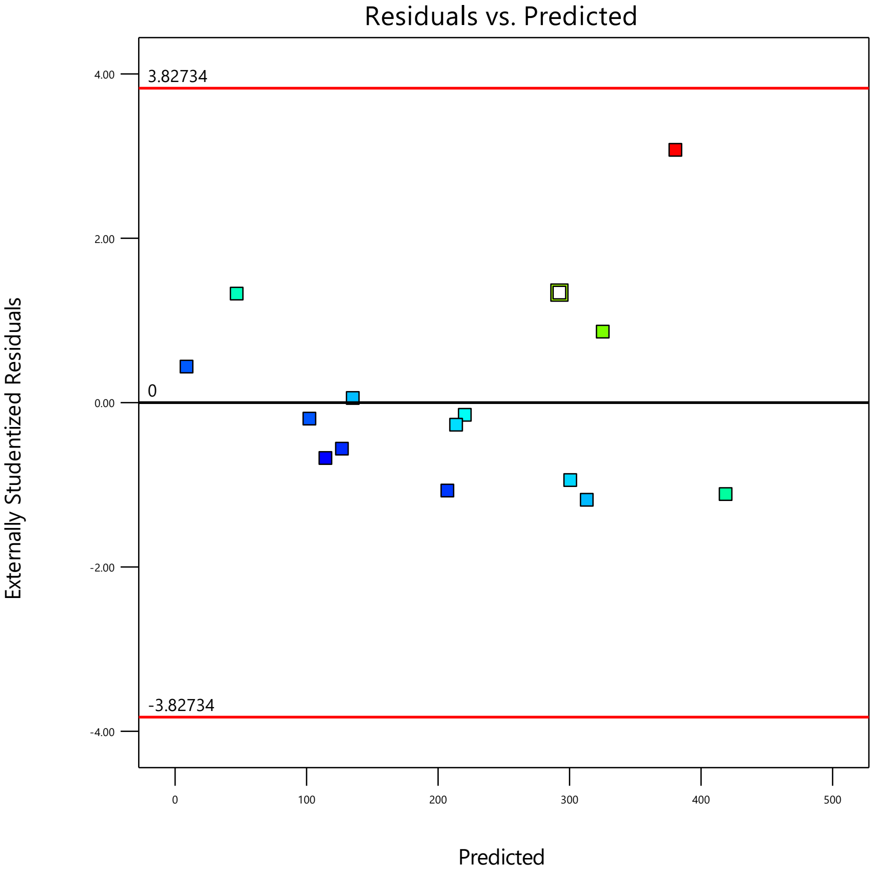

Figure S2. Normal Probability Plot and Residuals vs. Predicted Plot for the Round Response in RFB printed samples

Response: Round

Color points by value:

Round:

0.969 0.988

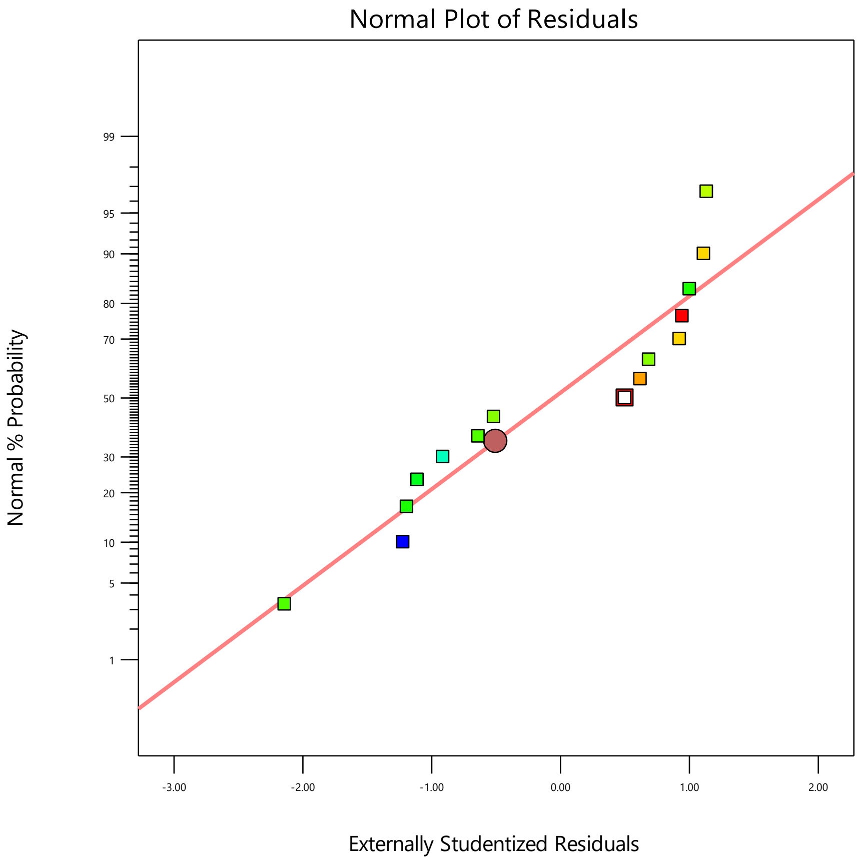

Response: Round

Color points by value:

Round:

0.969 0.988

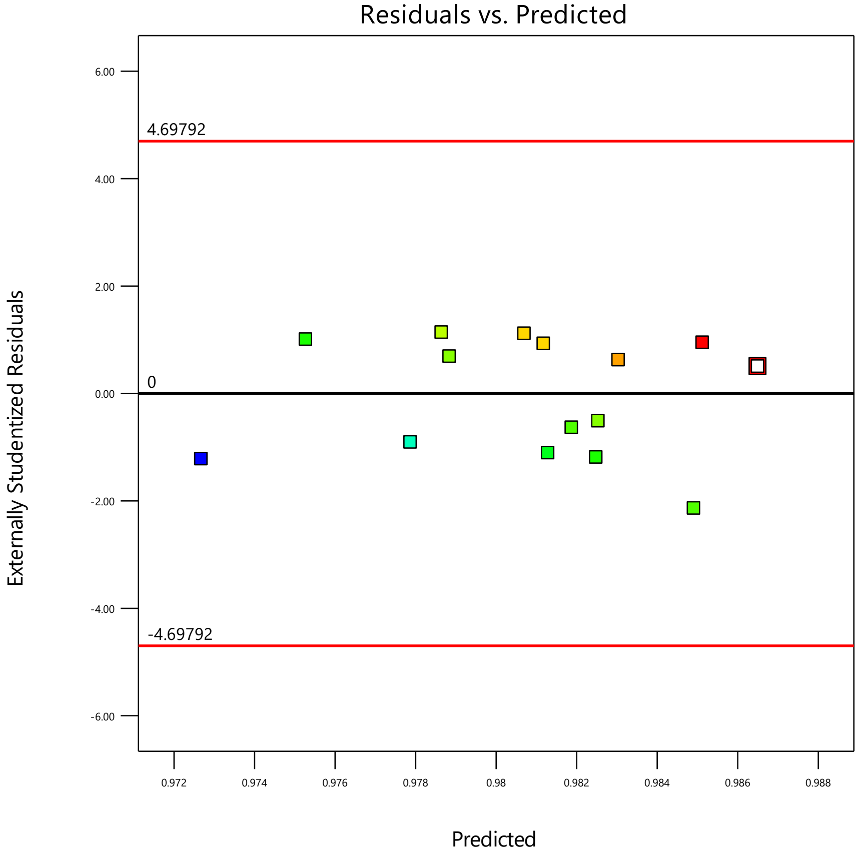

Figure S3. Normal Probability Plot and Residuals vs. Predicted Plot for the ASM Response in RFB printed samples

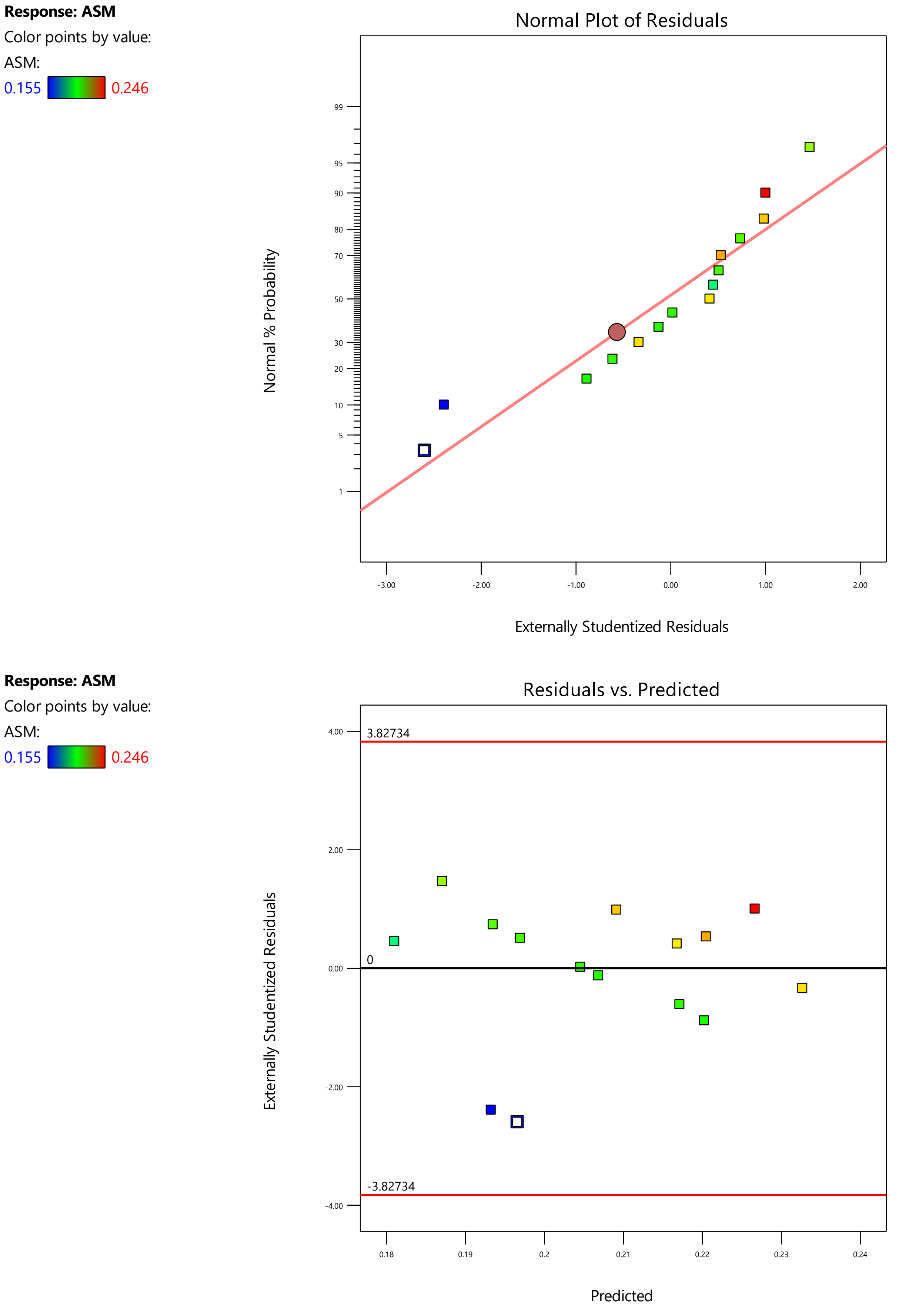

Figure S4. Normal Probability Plot and Residuals vs. Predicted Plot for the Entropy Response in RFB printed samples

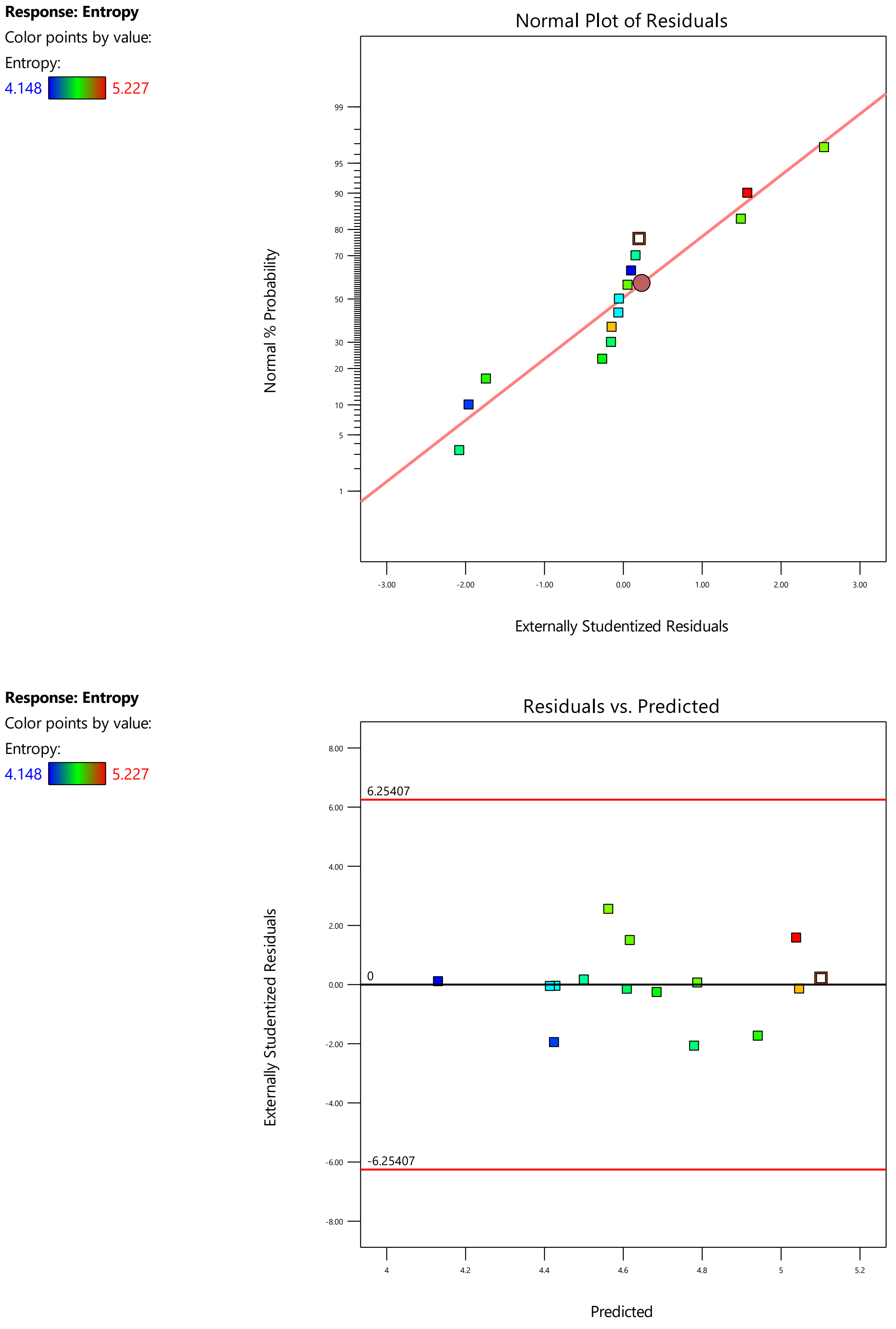

Figure S5. Normal Probability Plot and Residuals vs. Predicted Plot for the Firmness Response in RFB printed samples

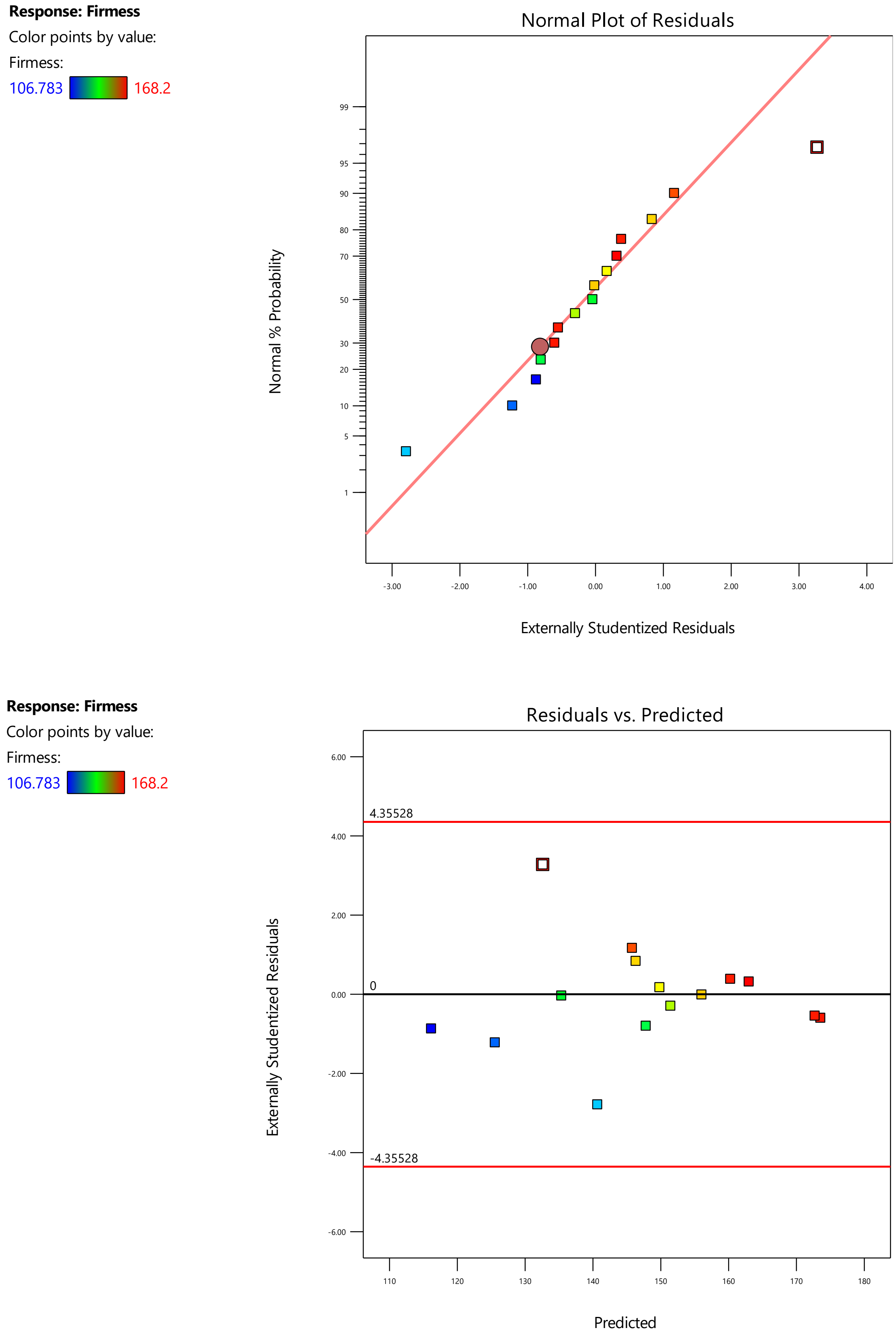

Figure S6. Normal Probability Plot and Residuals vs. Predicted Plot for the Cohesiveness Response in RFB printed samples

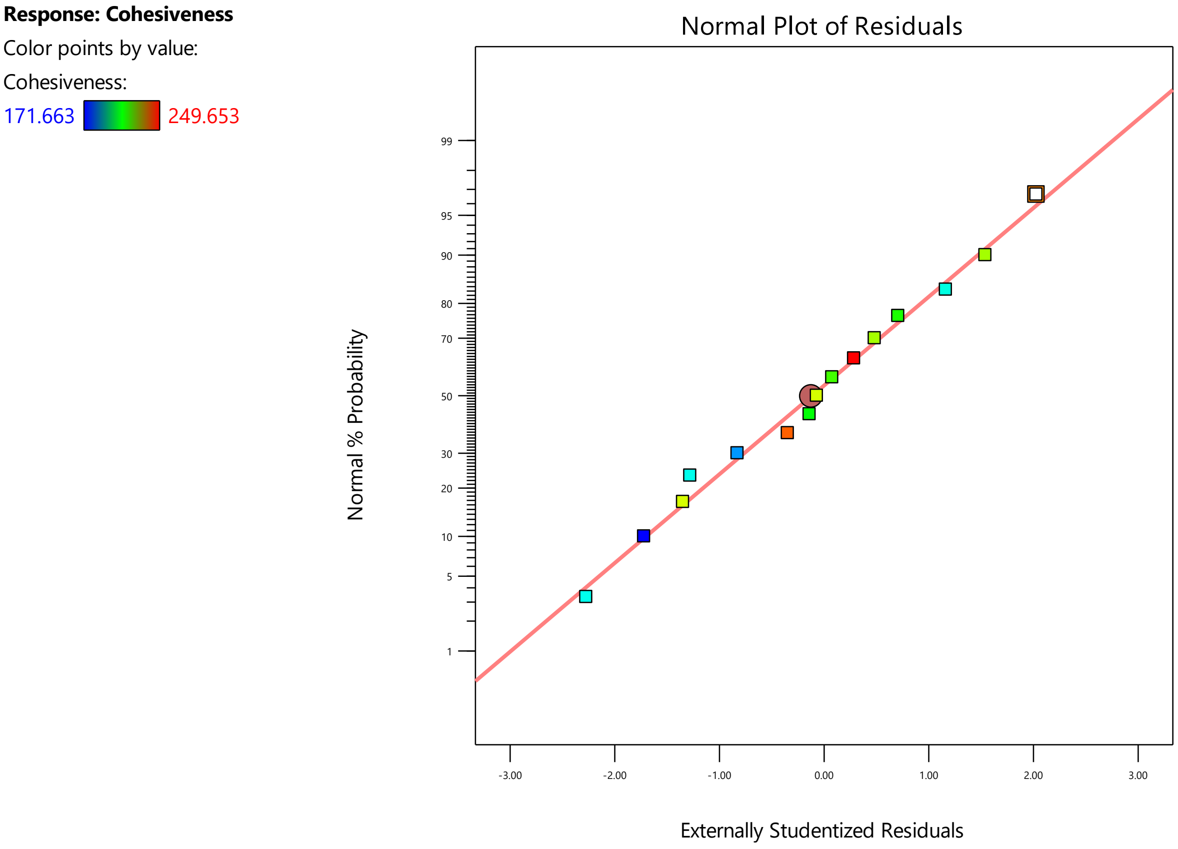

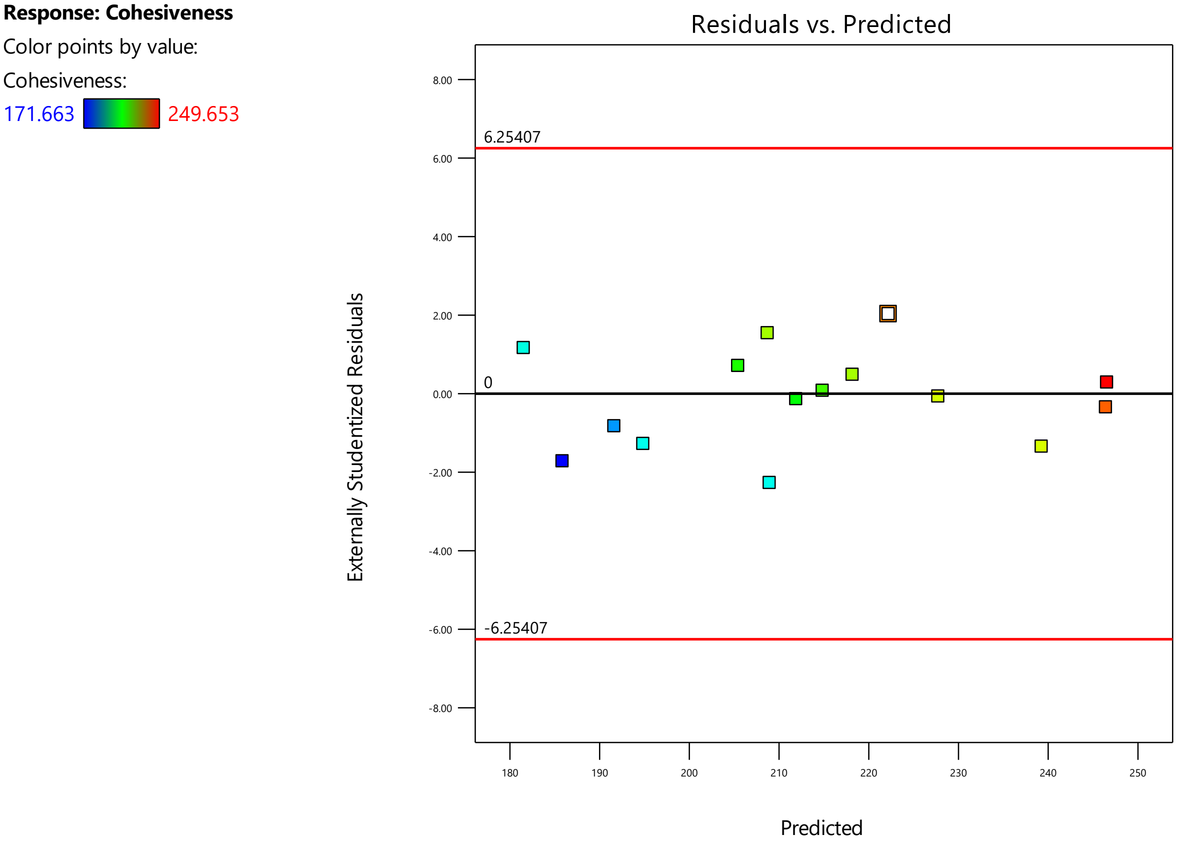

Figure S7. Normal Probability Plot and Residuals vs. Predicted Plot for the Discrepancy Area Response in EFB printed samples

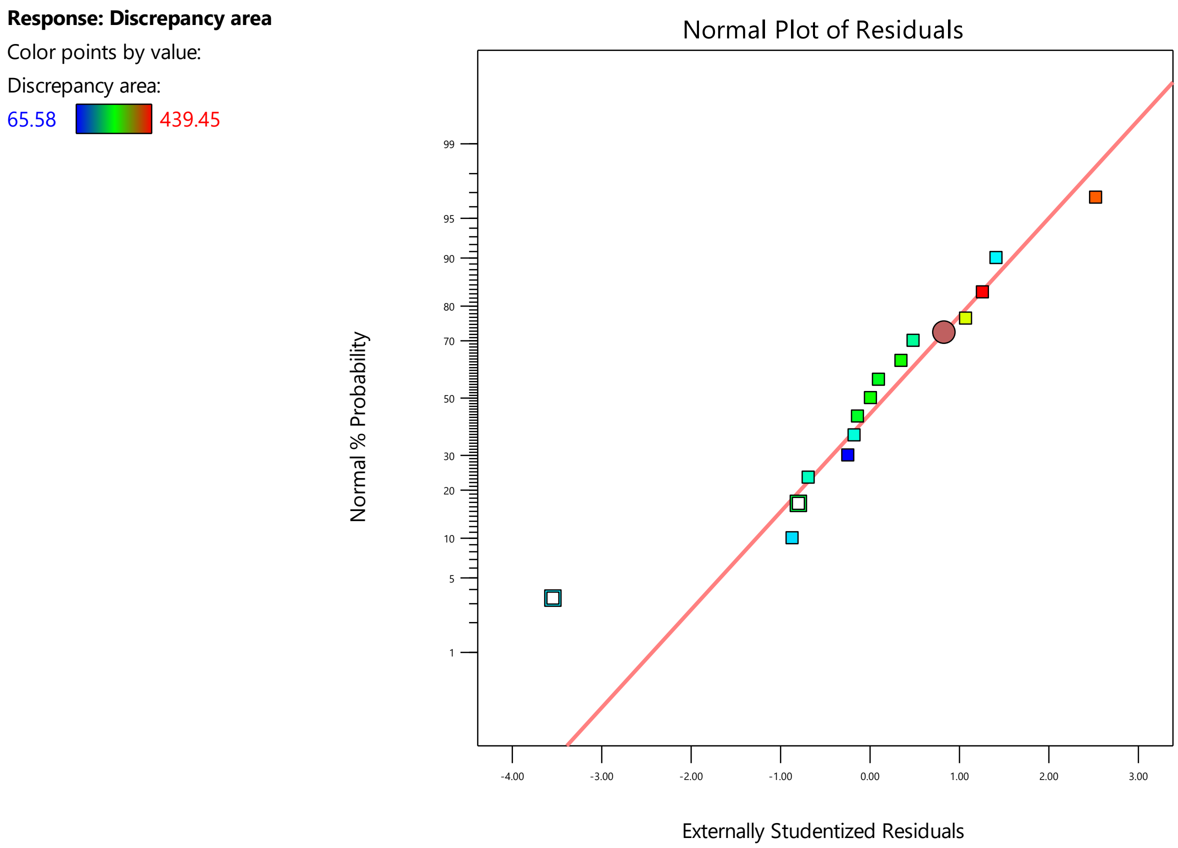

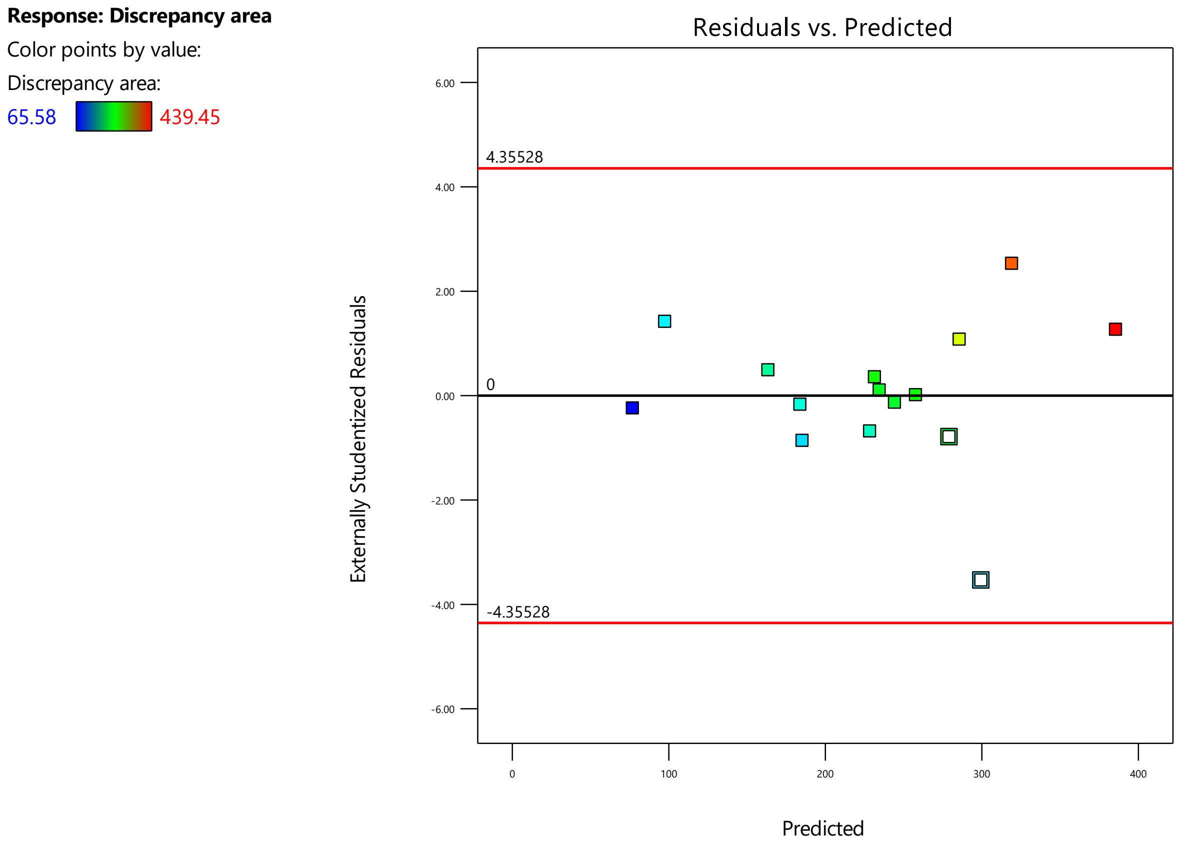

Figure S8. Normal Probability Plot and Residuals vs. Predicted Plot for the Perimeter Response in EFB printed samples

Response: Perimeter

Color points by value:

Perimeter:

242.982 276.394

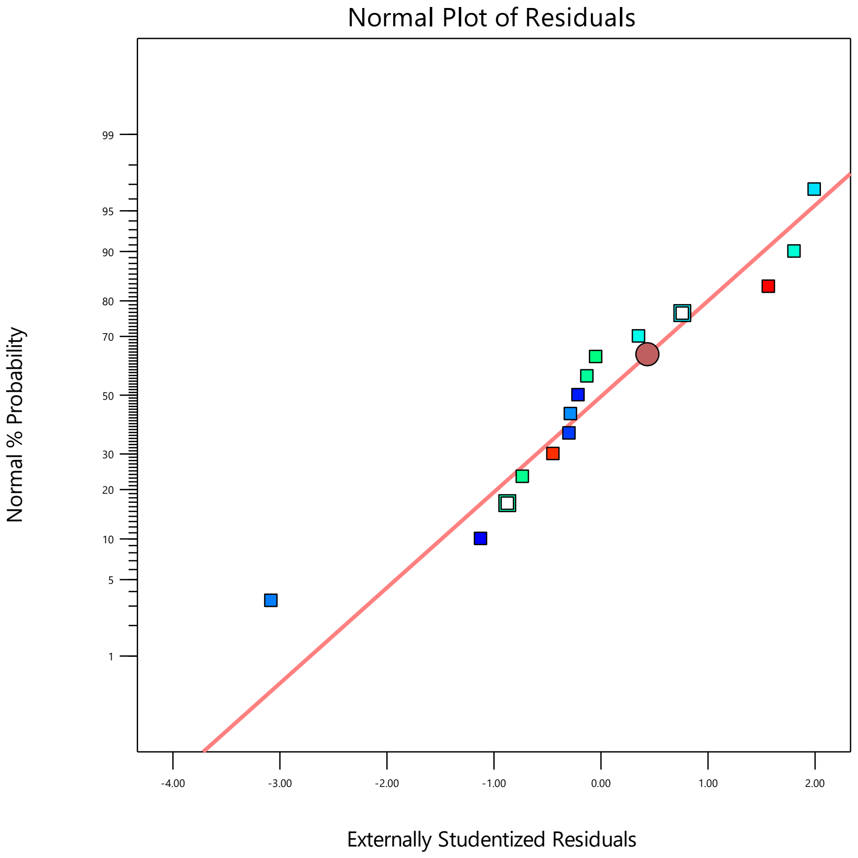

Response: Perimeter

Color points by value:

Perimeter:

242.982 276.394

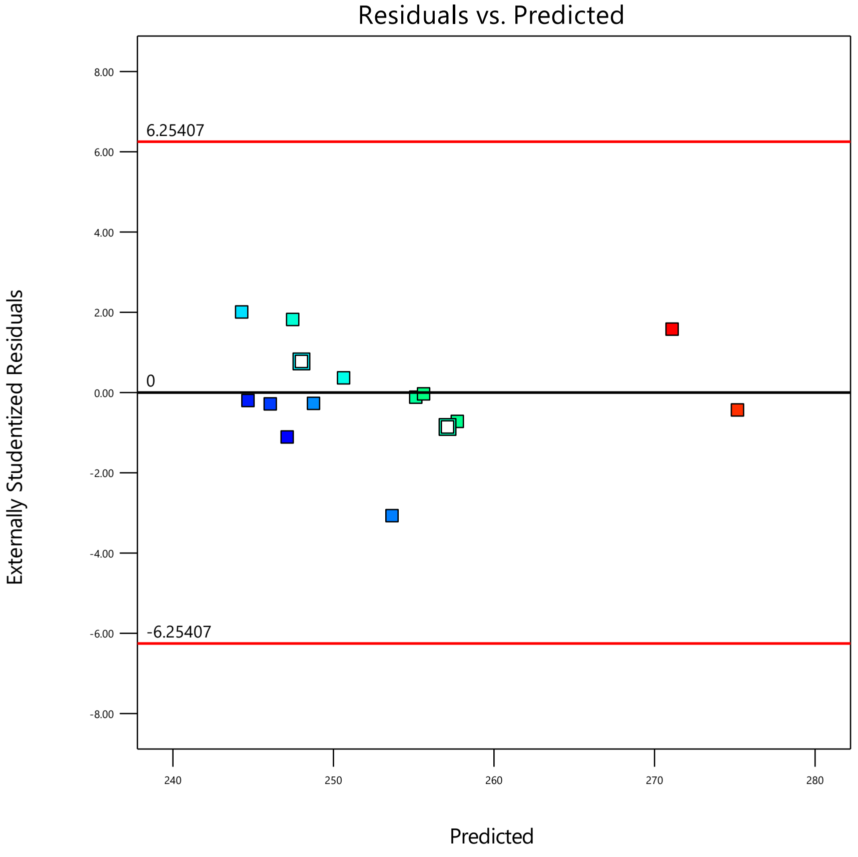

Figure S9. Normal Probability Plot and Residuals vs. Predicted Plot for the Circularity Response in EFB printed samples

Response: Circularity

Color points by value:

Circularity:

0.436 – 0.579

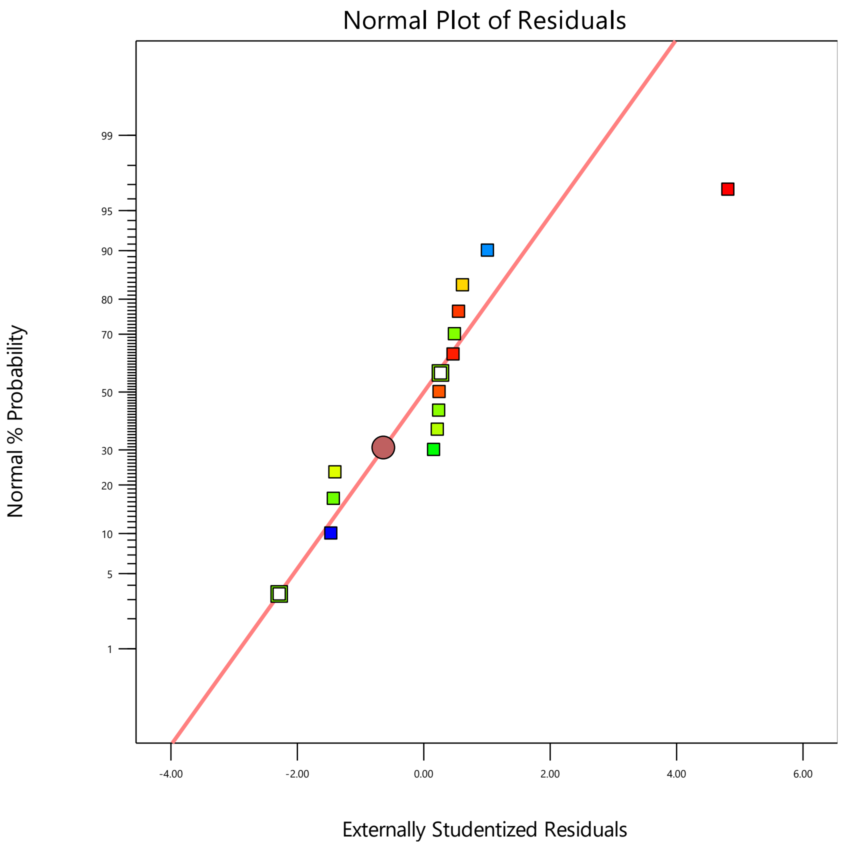

Response: Circularity

Color points by value:

Circularity:

0.436 – 0.579

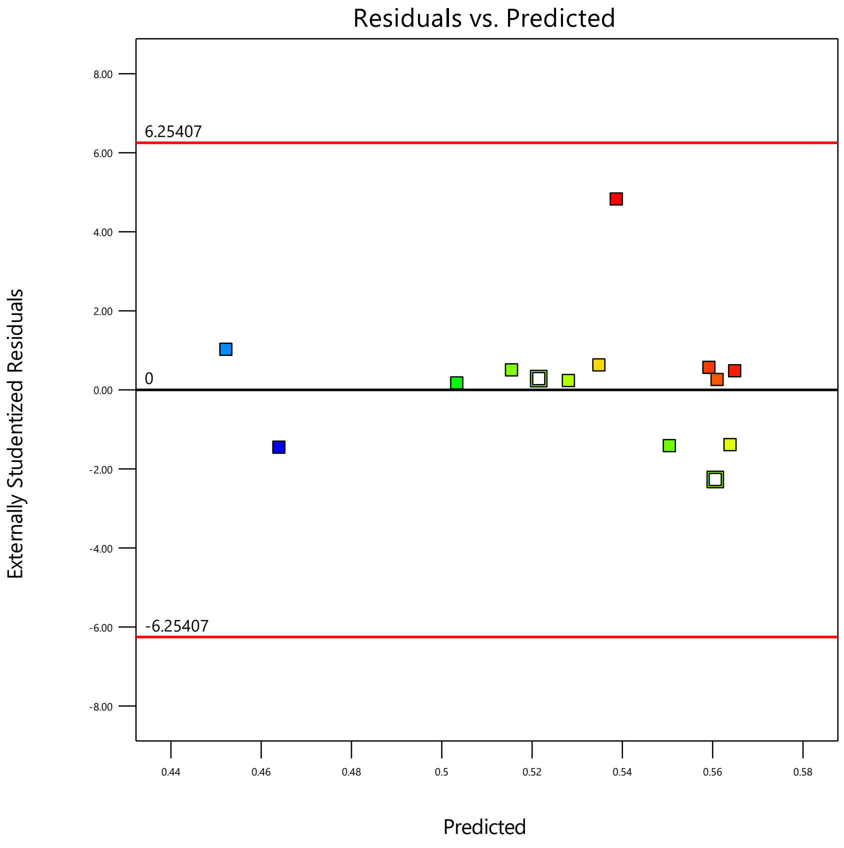

Figure S10. Normal Probability Plot and Residuals vs. Predicted Plot for the Round Response in EFB printed samples

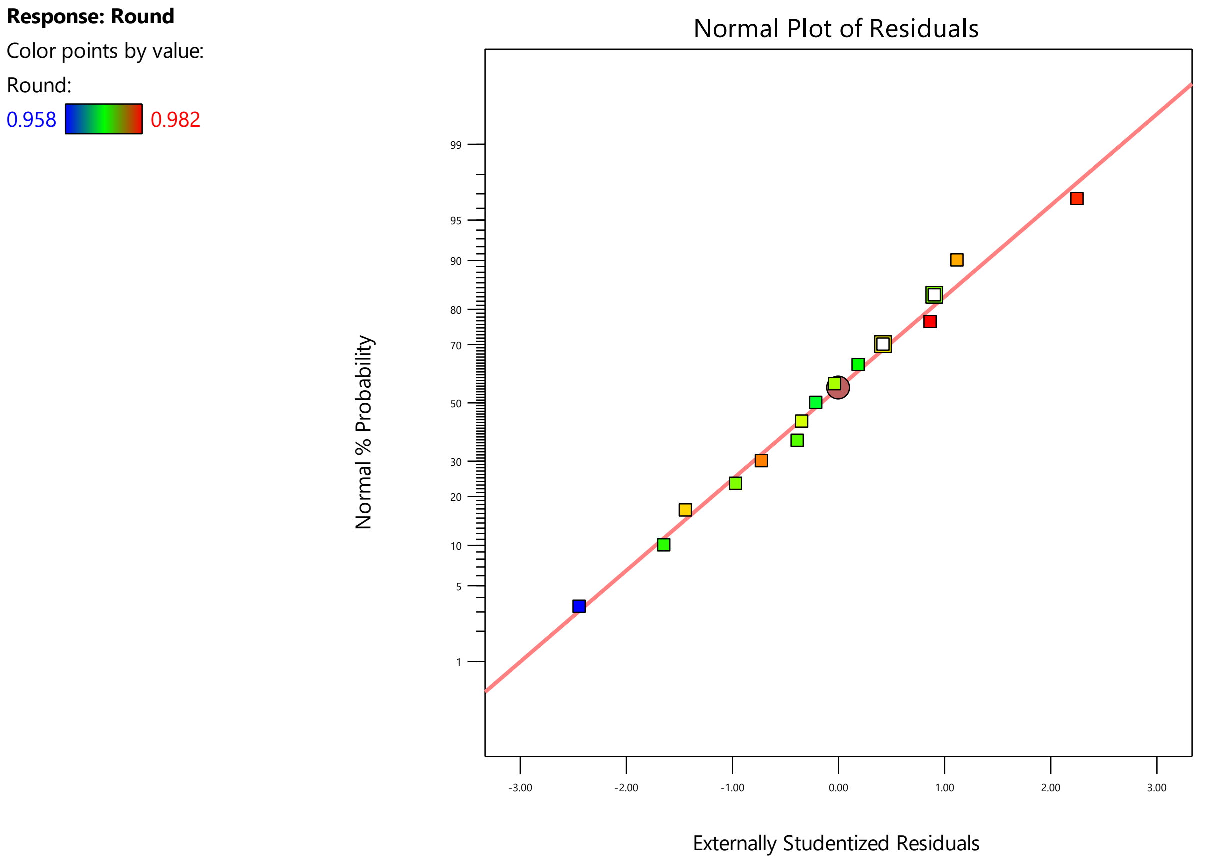

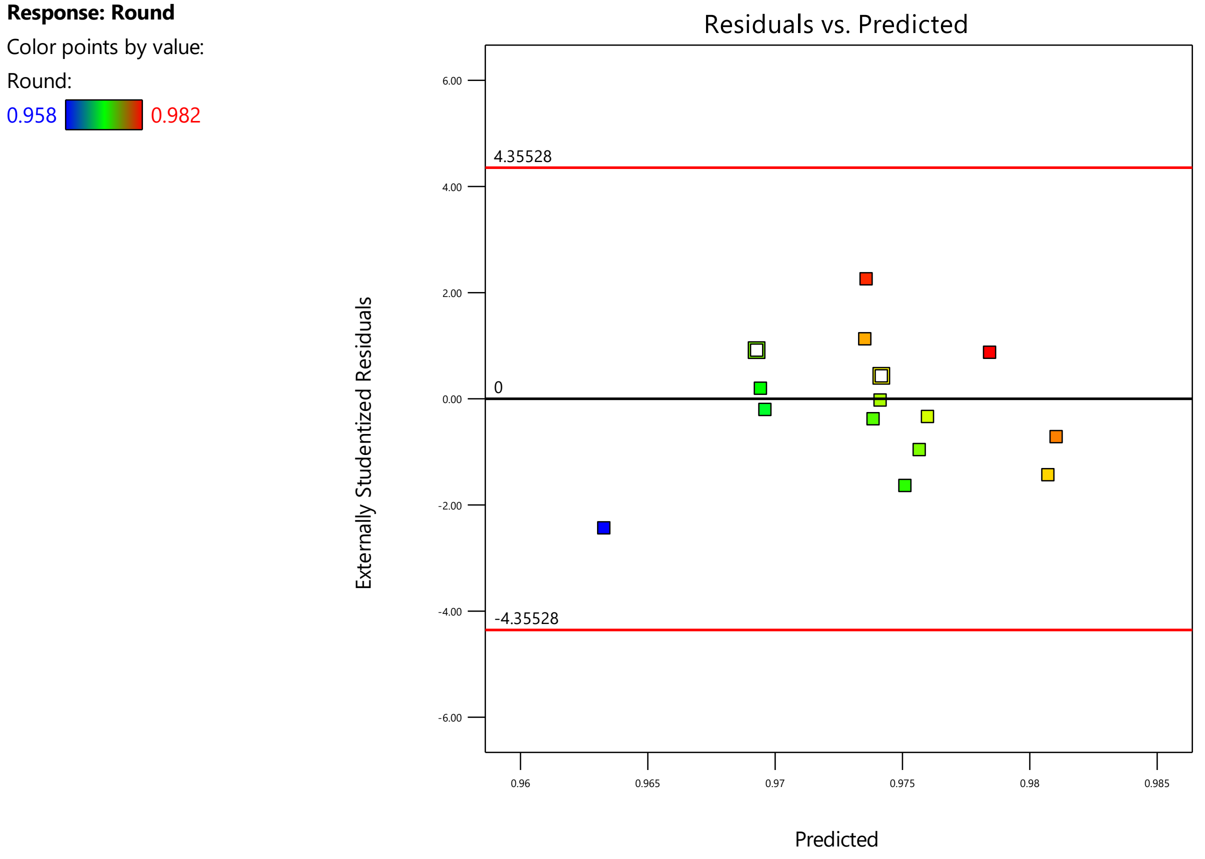

Figure S11. Normal Probability Plot and Residuals vs. Predicted Plot for the Entropy Response in EFB printed samples

Response: Entropy

Color points by value:

Entropy:

4.31 4.91

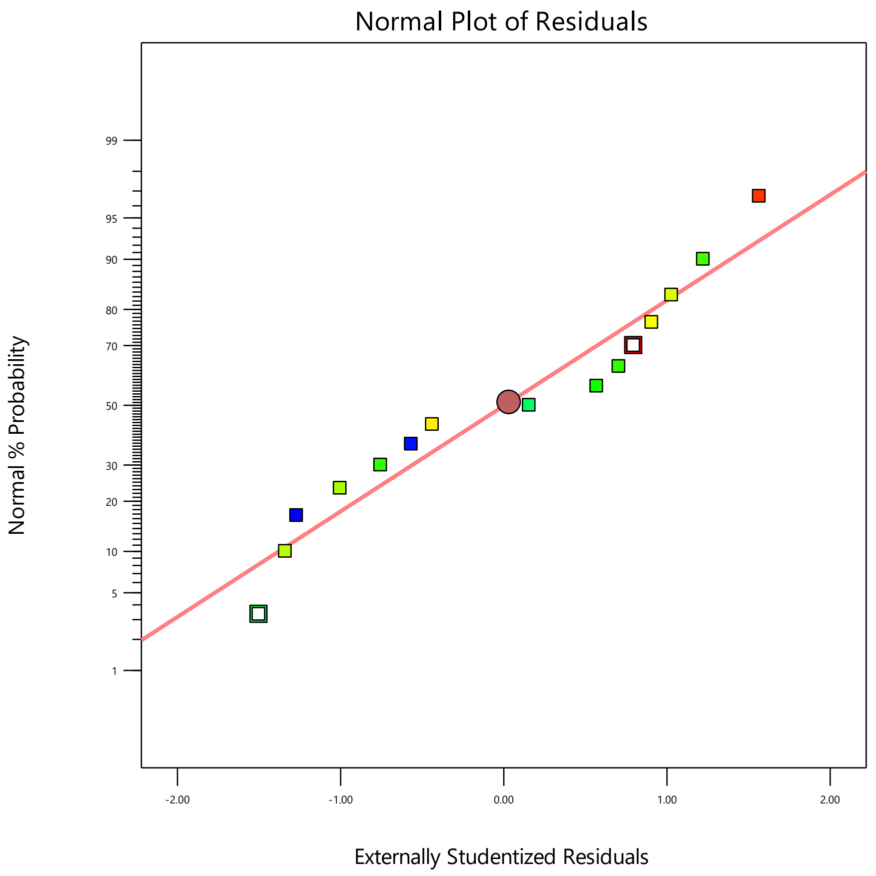

Response: Entropy

Color points by value:

Entropy:

4.31 4.91

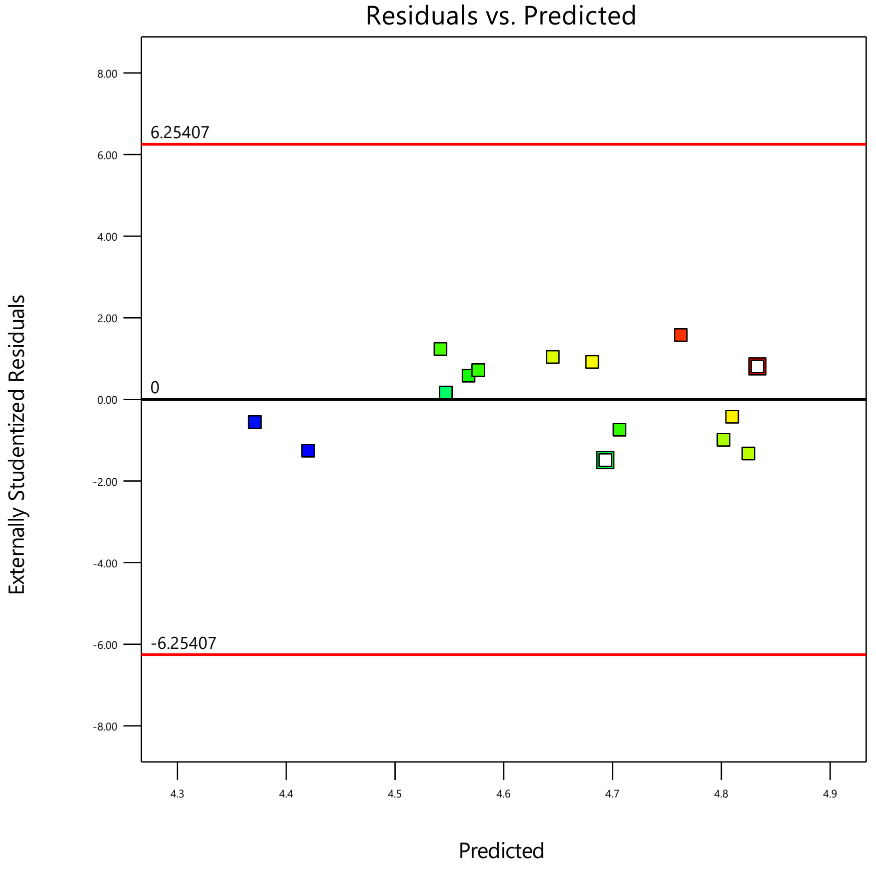

Figure S12. Normal Probability Plot and Residuals vs. Predicted Plot for the Firmess Response in EFB printed samples

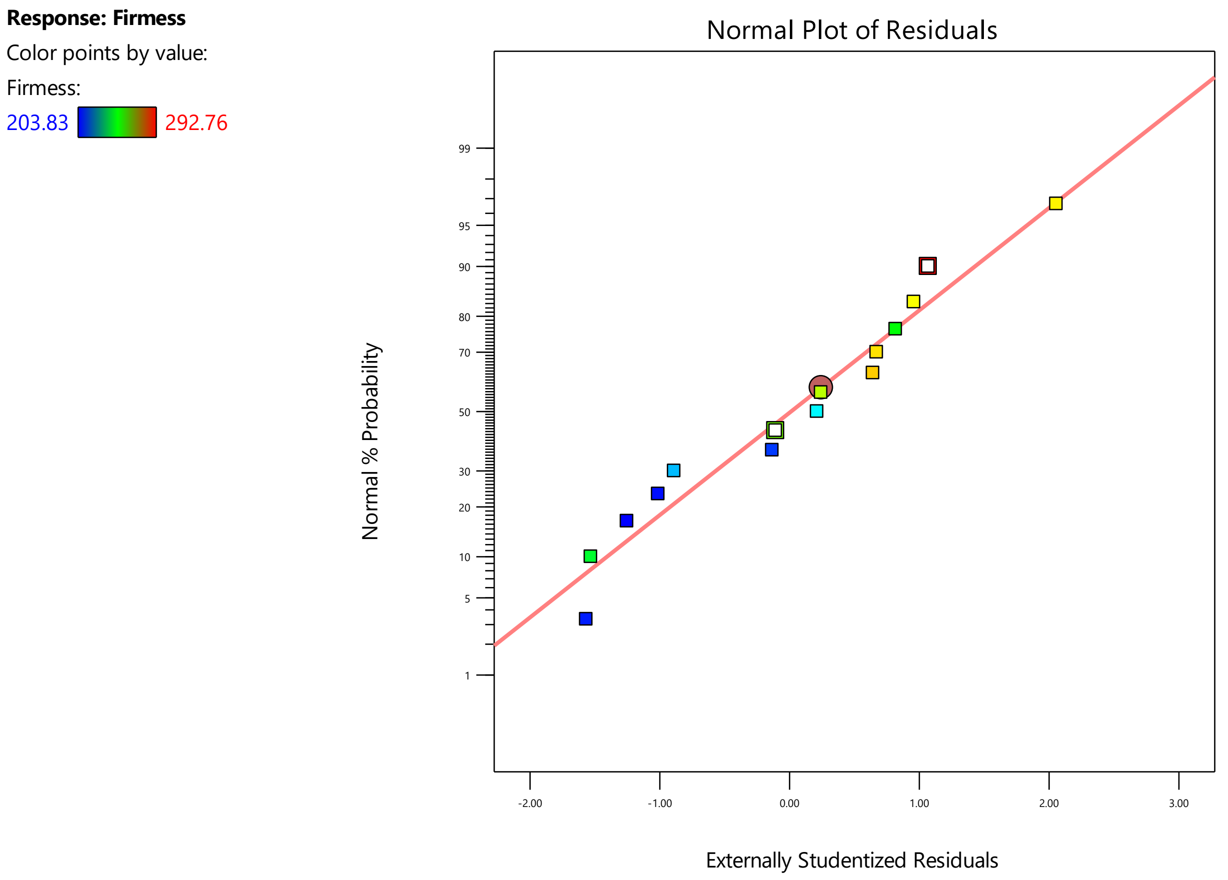

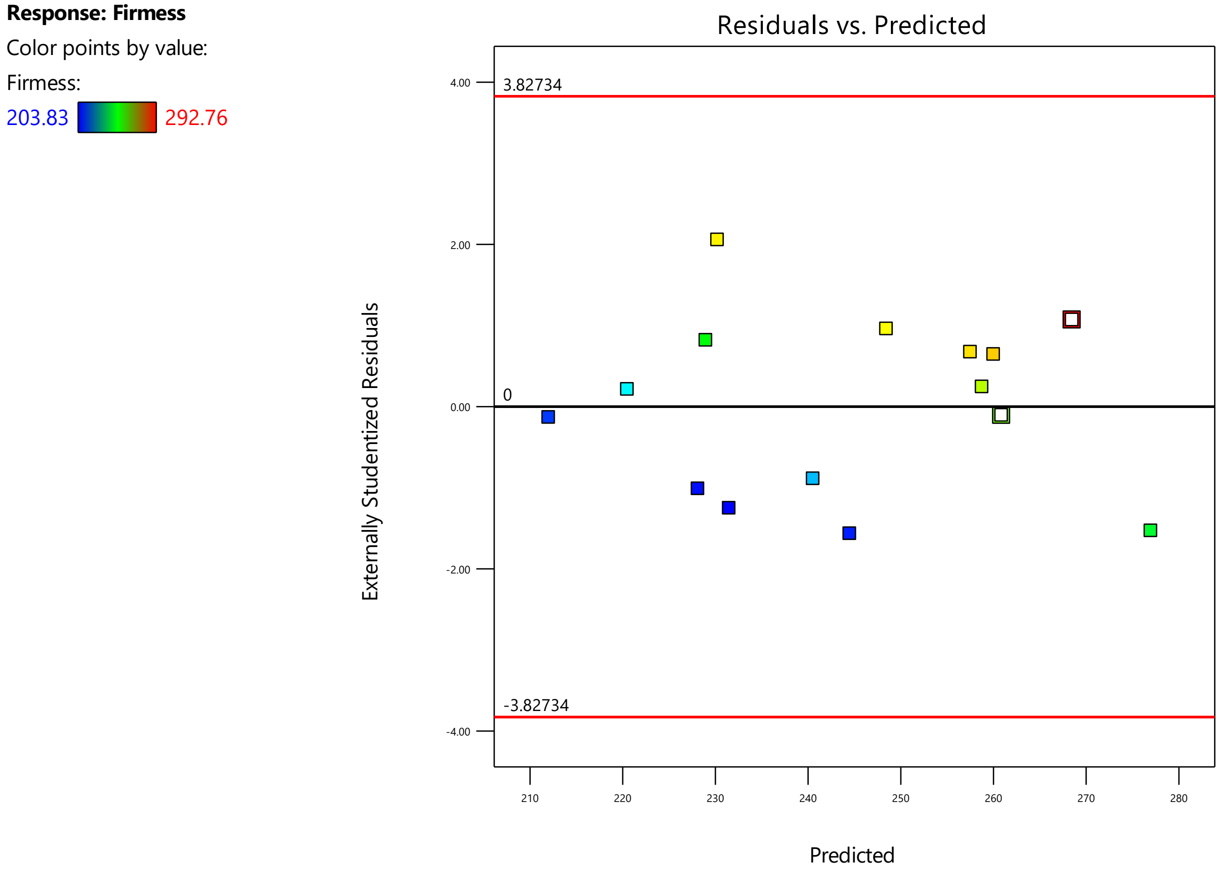

Supplement: Supplementary file 1 [file foods-14-00715-s001.zip › foods-3445511-supplementary.pdf]
